# Molecular Mechanisms and Multi-Omics Integration in Heart Failure: From Pathophysiology to Precision Medicine

**DOI:** 10.3390/ijms27114814

**Published:** 2026-05-27

**Authors:** Carlo Domenico Maida, Gaetano Pacinella, Mario Daidone, Mariarita Margherita Bona, Stefania Scaglione, Rachele Malfitano, Rosario Norrito, Giuliano Cassataro, Luigi Dell’Ajra, Sergio Ferrantelli, Gabriele Angelo Vassallo, Antonino Tuttolomondo

**Affiliations:** 1 Department of Internal Medicine, S. Elia Hospital, 93100 Caltanissetta, Italy; 2 Molecular and Clinical Medicine PhD Program, University of Palermo, 90133 Palermo, Italy; pacinella66@gmail.com (G.P.);; 3 Internal Medicine and Stroke Care Ward, Department of Health Promotion, Mother and Child Care, Internal Medicine and Medical Specialities, University of Palermo, 90127 Palermo, Italy; 4 Department of Internal Medicine, Barone Lombardo Hospital, 92024 Canicattì, Italy; rosario94.norrito@gmail.com; 5 Medicine Unit, Fondazione G. Giglio, 90015 Cefalù, Italy; giuliano.cassat@gmail.com; 6 Department of Internal Medicine, Buccheri La Ferla Hospital, 90123 Palermo, Italy

**Keywords:** heart failure, molecular remodelling, epigenetics, non-coding RNAs, proteomics, metabolomics, precision medicine, translational medicine

## Abstract

Heart failure (HF) is a complex and heterogeneous clinical syndrome defined by progressive structural, functional, and molecular alterations in the myocardium, representing a significant global health challenge. Beyond haemodynamic compromise, HF arises from intricate interactions among neurohormonal activation, chronic inflammation, oxidative stress, mitochondrial dysfunction, impaired calcium handling, and extracellular matrix remodelling. These processes drive maladaptive cardiac remodelling and progressive functional decline across multiple HF phenotypes, including HF with reduced (HFrEF), mildly reduced (HFmrEF), and preserved ejection fraction (HFpEF). Recent advances in molecular biology have highlighted the critical roles of genomic, epigenetic, and transcriptomic mechanisms in the progression of HF. DNA methylation, histone modifications, chromatin remodelling, and non-coding RNAs regulate gene expression in response to environmental and metabolic stimuli, thereby connecting systemic risk factors to cardiac dysfunction. Proteomic and post-translational modifications, such as phosphorylation, acetylation, and redox signalling, modulate protein function and contribute to contractile impairment and metabolic dysregulation. Metabolomic studies have revealed significant changes in myocardial energy metabolism, including reduced oxidative capacity, decreased metabolic flexibility, and limited bioenergetic reserves. The integration of multi-omics approaches—including genomics, transcriptomics, proteomics, metabolomics, and epigenomics—has provided unprecedented insight into the biological heterogeneity of HF, facilitating the identification of distinct molecular subtypes and novel therapeutic targets. Systems biology and network-based analyses, supported by artificial intelligence and machine learning, enable the synthesis of complex datasets and enhance risk classification, prognosis, and personalised treatment approaches. This narrative review synthesises the current understanding of the molecular mechanisms underlying HF, with particular emphasis on the interplay between metabolic and epigenetic regulation in disease progression. It also highlights emerging translational opportunities, including omics-based biomarkers, targeted therapies, and precision medicine approaches. Despite significant advances, challenges remain in translating these findings into clinical practice, underscoring the need for standardised methodologies, extensive validation, and integrative frameworks. Ultimately, a systems-level, multi-omics perspective is crucial for redefining HF as a biologically stratified condition in the landscape of advancing tailored cardiovascular medicine.

## 1. Introduction

Heart failure (HF), as defined by the American College of Cardiology (ACC) and the American Heart Association (AHA), is a complex clinical syndrome resulting from structural and/or functional impairment of ventricular filling or blood ejection.

HF is a serious global health problem that affects an estimated 55–64 million individuals worldwide, with a prevalence of approximately 1–3% in the general adult population, contributing significantly to morbidity, mortality, healthcare costs, and deterioration in quality of life. The aetiology of HF is heterogeneous and multifactorial, with ischaemic heart disease being the leading cause globally [1].

Further highlighting the growing impact of HF, the Global Burden of Disease (GBD) 2021 study reported that global prevalence has more than doubled from 1990 to 2021. This increase depends strongly on age: fewer than 2% of people younger than 60 are affected, rising to over 10% in those aged 75 or older [2,3].

The escalating global burden of HF highlights the need to understand its biological mechanisms. Its age dependency reflects cumulative cardiovascular risk factors, comorbidities, and structural cardiac changes over time. These epidemiological patterns intertwine with pathophysiological processes, such as maladaptive neurohormonal activation, ventricular remodelling, impaired myocardial energetics, and systemic inflammation. Understanding the interplay among these mechanisms is essential to connect population-level observations with disease biology, guiding more effective strategies for prevention, early detection, and targeted intervention.

HF is a complex and progressive syndrome arising from the dynamic interplay of neurohormonal, structural, cellular, and metabolic alterations that ultimately impair cardiac performance. A key initiating and perpetuating mechanism is chronic neurohormonal activation. In response to reduced cardiac output, sustained activation of the sympathetic nervous system and the renin–angiotensin–aldosterone system initially preserves perfusion. However, over time, this compensatory response becomes maladaptive, promoting β-adrenergic receptor desensitisation, vasoconstriction, sodium retention, and profibrotic signalling, thereby establishing a self-reinforcing cycle of functional deterioration [4]. These neurohormonal changes drive cardiac remodelling, a process characterised by profound alterations in myocardial structure and gene expression. Cardiomyocytes undergo hypertrophy and reprogramme to a foetal transcriptional programme, while progressive cell loss through apoptosis and necrosis, along with dysregulated autophagy, contribute to ventricular dilation and worsening contractile function. In parallel, fibroblast activation and extracellular matrix deposition increase myocardial stiffness [5]. At the cellular level, defective calcium handling represents a central mechanism linking molecular alterations to impaired mechanical performance. Reduced sarcoplasmic reticulum calcium reuptake, abnormal ryanodine receptor function, and diminished β-adrenergic responsiveness result in both systolic dysfunction and impaired relaxation, while also predisposing to arrhythmias [6]. Superimposed on these processes is a state of mitochondrial dysfunction and energetic deficit. Impaired oxidative phosphorylation and altered mitochondrial calcium signalling reduce (Adenosine Triphosphate) ATP availability, compromising the energy supply required for contraction and ionic homeostasis. Excessive production of reactive oxygen species further exacerbates cellular injury and maladaptive signalling [7]. Finally, changes in sarcomeric and cytoskeletal proteins, particularly titin, alter the myocardium’s intrinsic mechanical properties. Modifications in titin phosphorylation and isoform expression contribute to increased passive stiffness, especially in forms of HF characterised by preserved ejection fraction [8]. Collectively, these tightly interconnected mechanisms transform an initially adaptive response into a maladaptive process, driving the progression of HF toward a state of reduced efficiency, impaired reserve, and clinical decompensation.

Clinically, HF may also be broadly classified based on predominant ventricular involvement as left-sided, right-sided, or biventricular HF. Left-sided HF is characterised by pulmonary congestion and impaired systemic perfusion due to left ventricular systolic or diastolic dysfunction. In contrast, right-sided HF is typically associated with systemic venous congestion, elevated pulmonary pressures, pulmonary vascular disease, or chronic left-sided HF. These phenotypes often overlap and share molecular mechanisms, including neurohormonal activation, endothelial dysfunction, inflammation, mitochondrial impairment, and maladaptive remodelling [4,5,6,7,8]. Beyond ischaemic and primary myocardial diseases, HF may also arise secondary to a wide range of systemic and extracardiac conditions. Metabolic disorders, including obesity, insulin resistance, and diabetes mellitus, contribute to myocardial dysfunction through chronic inflammation, endothelial impairment, mitochondrial dysfunction, and altered substrate utilisation. These mechanisms are particularly relevant in heart failure with preserved ejection fraction (HFpEF). Persistent tachyarrhythmias, such as atrial fibrillation and tachycardia-mediated cardiomyopathy, can further promote adverse remodelling and progressive ventricular dysfunction through sustained neurohormonal activation and impaired calcium handling [6]. Pulmonary vascular and pericardial diseases may also contribute to right-sided HF by increasing right ventricular afterload and impairing ventricular filling. Additionally, drug- or toxin-induced myocardial injury can directly induce oxidative stress, mitochondrial dysfunction, inflammation, and regulated cardiomyocyte death [7]. Despite their diverse origins, these conditions frequently converge on shared molecular pathways involving fibrosis, endothelial dysfunction, metabolic remodelling, and maladaptive neurohormonal signalling.

**Figure 1:**
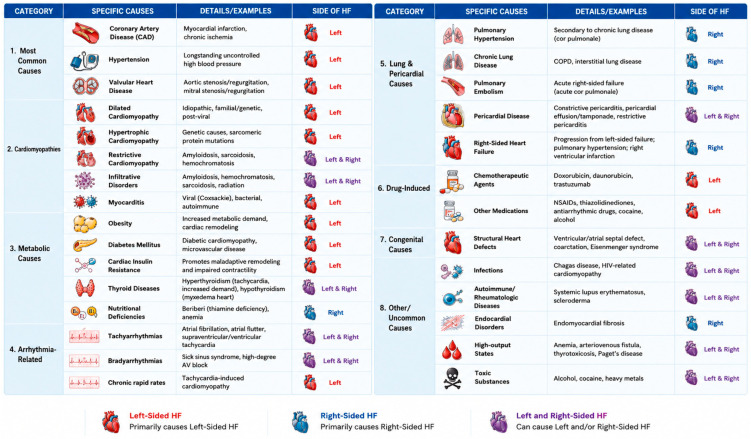
Pathogenic categories of heart failure, along with their associated clinical aspects and underlying mechanisms.

HF results from a broad spectrum of underlying pathophysiological processes that often overlap among different etiological categories. Identifying the primary cause is essential, as it enables clinicians to implement targeted, aetiology-specific therapeutic strategies that can significantly affect disease prognosis and progression (Figure 1).

HF is characterised by a range of maladaptive molecular and cellular processes, including neurohormonal overactivation, impaired calcium handling, mitochondrial dysfunction, oxidative stress, and remodelling of the extracellular matrix and sarcomeric proteins. HFrEF is mainly driven by cardiomyocyte loss, ventricular dilation, and overt systolic dysfunction, whereas HFmrEF displays intermediate patterns of partial systolic impairment and mixed remodelling. In contrast, HFpEF features preserved systolic function alongside pronounced diastolic dysfunction, increased myocardial stiffness, and subtle cardiomyocyte and microvascular abnormalities. These shared molecular changes impair myocardial efficiency and reserve, providing a substrate upon which systemic influences—including neurohumoral signals driven by central nervous system (CNS) dysregulation, as well as metabolic and inflammatory signals from adipose tissue, the kidneys, and skeletal muscle—exert profound modulatory effects. Although the cellular and molecular mechanisms of HFpEF are less defined, evidence and models increasingly implicate myocardial fibrosis, chronic inflammation, microvascular dysfunction, disrupted nitric oxide-cGMP signalling, altered energy metabolism, and mitochondrial abnormalities as central contributors to its pathophysiology [9].

This review integrates current knowledge on the molecular and epigenetic basis of HF with a focus on the complex, dynamic interplay between signalling pathways, metabolic remodelling, and gene regulatory networks that drive disease progression. Despite significant advances, the mechanistic links connecting these processes remain incompletely understood. To address these knowledge gaps, this review specifically focuses on the mechanistic interplay among neurohormonal activation, metabolic remodelling, inflammation, mitochondrial dysfunction, and gene regulatory networks that drive HF progression. In parallel, we explore epigenetic mechanisms, such as DNA methylation, histone modifications, chromatin architecture, and noncoding RNAs, which act as key modulators of transcriptional reprogramming in the failing heart.

This manuscript also focuses on the interplay between metabolic state and epigenetic regulation, emphasising how metabolic intermediates directly influence chromatin-modifying enzymes and gene expression. By framing HF within this context, we aim to identify critical knowledge gaps and offer a conceptual framework for future research and therapeutic innovation.

## 2. Molecular and Cellular Remodelling in Heart Failure

Heart failure is a complex and multifactorial clinical syndrome characterised by significant structural, cellular, and molecular alterations of the myocardium. Emerging evidence suggests that cardiac remodelling in this context is not governed by isolated pathways but results from the dynamic interaction of multiple, closely interconnected biological processes. Cellular and molecular remodelling in heart failure can therefore be conceptualised as occurring along four integrated and interdependent axes.

A comprehensive understanding of these axes, and particularly their functional integration, is essential for elucidating the underlying pathophysiological mechanisms and advancing beyond reductionist interpretations of the disease. Integrating these dimensions may establish the foundation for a novel prognostic and therapeutic paradigm, thereby enabling more precise and personalised strategies for the management of heart failure.

### 2.1. Neurohormonal–Metabolic Crosstalk

Neurohormonal activation acts as a fundamental mechanism in the pathophysiology of HF, largely independent of ejection fraction. When cardiac output becomes insufficient to maintain adequate tissue perfusion, an evolutionarily conserved compensatory response is initiated. This response involves the sympathetic nervous system (SNS), the renin–angiotensin–aldosterone system (RAAS), and arginine vasopressin (AVP). It is mainly triggered by arterial underfilling, defined as a reduction in effective arterial blood volume. High-pressure baroreceptors in the left ventricle, carotid sinus, and aortic arch sense this change. Arterial underfilling can occur in low-output HF, in which reduced cardiac output directly reduces arterial filling. It can also arise in high-output HF, which is characterised by peripheral vasodilation and decreased systemic vascular resistance that leads to relative hypoperfusion. In acute settings, the SNS provides the fastest compensatory response. Increased sympathetic tone enhances heart rate and myocardial contractility to offset reduced stroke volume. Systemic vasoconstriction preserves perfusion pressure to critical organs. At the same time, increased venous tone augments venous return and preload. Sympathetic stimulation of the kidneys promotes renin release, activating the RAAS cascade. Angiotensin II is a potent vasoconstrictor, enhances myocardial contractility, and stimulates aldosterone secretion. Aldosterone promotes renal sodium reabsorption and intravascular volume expansion. In parallel, AVP is released in response to non-osmotic stimuli. It acts via V1a receptors to induce vasoconstriction and via V2 receptors to increase renal water reabsorption. These mechanisms are highly effective in the short term. They preserve perfusion and secure survival during haemodynamic stress [10,11,12].

However, with persistent activation, these initially adaptive reactions become maladaptive and drive disease progression. Specifically, chronic SNS stimulation exerts direct cardiotoxic effects via sustained catecholamine exposure, which in turn promotes cardiomyocyte apoptosis, hypertrophy, and interstitial fibrosis. Furthermore, a self-sustaining cycle develops, characterised by heightened sympatho-excitatory and reduced sympatho-inhibitory reflexes, thereby perpetuating sympathetic overactivity [13].

Similarly, RAAS activation extends beyond its haemodynamic effects. Angiotensin II directly induces hypertrophy, apoptosis, and fibrotic remodelling in both cardiomyocytes and renal tubular cells. Chronically elevated aldosterone further increases tissue fibrosis by stimulating macrophage-mediated secretion of galectin-3, which activates fibroblasts and promotes collagen deposition in the myocardium and kidneys. In healthy individuals, a physiological “aldosterone escape” occurs; however, this response is impaired in HF, resulting in sustained sodium retention, volume overload, and congestion.

Aldosterone escape may contribute differently across heart failure (HF) phenotypes. In heart failure with reduced ejection fraction (HFrEF), persistent activation of the renin–angiotensin–aldosterone system (RAAS), sympathetic overactivity, and reduced renal perfusion sustain aldosterone production despite ACE inhibition or angiotensin receptor blockade. This process promotes sodium retention, adverse ventricular remodelling, and myocardial fibrosis. In contrast, in heart failure with preserved ejection fraction (HFpEF), aldosterone excess is more closely associated with obesity, systemic inflammation, endothelial dysfunction, and coronary microvascular impairment. In this context, mineralocorticoid receptor overactivation primarily contributes to myocardial stiffness, diastolic dysfunction, and interstitial fibrosis. These phenotype-specific mechanisms may help explain the heterogeneous clinical responses observed with mineralocorticoid receptor antagonists in different HF populations.

Elevated AVP levels also contribute to maladaptive remodelling. Specifically, AVP directly promotes hypertrophic signalling and worsens fluid retention via persistent V2 receptor activation, which leads to congestion and hyponatremia—a well-established marker of poor prognosis. Additionally, AVP may weaken β-adrenergic receptor responsiveness, further compromising myocardial contractility [14,15].

Although neurohormonal activation is a hallmark of all HF phenotypes, its magnitude and profile vary significantly. In HFrEF, activation is typically more pronounced and homogeneous. Circulating levels of renin, aldosterone, norepinephrine, and natriuretic peptides are markedly elevated. Only 10% of patients with HFpEF have simultaneous elevations in renin, aldosterone, and norepinephrine, compared with 21% in patients with HFrEF [16].

By contrast, in HFpEF, neurohormonal activation is more heterogeneous and less uniformly expressed. Sympathetic activity appears to be preferentially directed toward the kidneys and the splanchnic circulation [17,18]. This reduces venous capacitance and increases the stressed blood volume. While aldosterone levels are often elevated and correlate with alterations in ventricular geometry, HFpEF is additionally characterised by increased leptin levels, relative suppression of natriuretic peptides, and resistance to natriuretic peptide signalling. This resistance may be mediated by enhanced neprilysin activity. This distinct neurohormonal profile reflects the predominant contribution of systemic inflammation, endothelial dysfunction, and metabolic dysregulation in HFpEF. It is not driven by impaired cardiac output alone [17,18].

Chronic neurohormonal activation creates a self-perpetuating vicious cycle. Increased preload and afterload, sodium and water retention, progressive fibrosis, and adverse ventricular remodelling further weaken cardiac function. This cycle worsens arterial underfilling and maintains neurohormonal drive. Circulating mediators rise directly with disease severity, even in asymptomatic left ventricular dysfunction. This pathophysiology underpins HF therapy, making pharmacological inhibition of neurohormonal systems a core treatment, especially in HFrEF.

Moreover, chronic neurohormonal stimulation promotes cardiac insulin resistance and abnormalities in substrate utilisation [19]. This creates a marked imbalance between catabolic and anabolic hormones, establishing a self-perpetuating cycle. As a result, metabolic dysfunction worsens cardiac impairment. This paradigm is exemplified by the low Triiodothyronine (T3) syndrome, which is partly due to increased myocardial type 3 deiodinase activity. This induces local hypothyroidism, impairing mitochondrial function and energy efficiency. Similarly, elevated parathyroid hormone levels and disruption of the GH–IGF-1 (growth hormone–insulin-like growth factor-1) axis are associated with disease severity and adverse outcomes. These changes are characterised by both deficiency and acquired resistance. A pronounced catabolic state, marked by increased cortisol and inflammatory cytokines, as well as reduced testosterone, DHEA (Dehydroepiandrosterone), and IGF-1, leads to skeletal muscle wasting and cardiac cachexia. This condition is strongly linked to mortality. Sex hormone imbalance further modulates disease expression in a context-dependent and sex-specific manner, particularly in HF with preserved ejection fraction. All these alterations converge to promote mitochondrial dysfunction, reduced ATP production, systemic inflammation, and proteolysis. Together, they reinforce a cycle of progressive cardiac and metabolic decline. Recognising HF as a syndrome of multisystem endocrine and metabolic imbalance has important therapeutic implications. It demonstrates the need for phenotype-driven strategies. These strategies should extend beyond the conventional neurohormonal blockade to target the underlying metabolic and hormonal abnormalities [20,21,22,23].

### 2.2. Inflammation and Endothelial Dysfunction

Inflammation represents a unifying pathophysiological feature across the spectrum of HF, yet its conceptual framework has undergone a fundamental shift from being viewed as purely deleterious to being recognised as a complex, context-dependent, and temporally dynamic immune response. The same signalling pathways may exert protective or harmful effects depending on the stage of the disease and the intensity and duration of activation, a phenomenon resembling that observed in neurohormonal signalling. Activation of the innate immune system in the failing heart occurs as a chronic, low-grade para-inflammatory response to ongoing tissue injury, mediated by pattern recognition receptors (PRRs), particularly Toll-like receptors (TLRs) and NOD-like receptors (NLRs). These receptors detect both damage-associated molecular patterns (DAMPs) released by stressed or injured cells and pathogen-associated molecular patterns (PAMPs) that may translocate across an impaired intestinal barrier resulting from intestinal hypoperfusion and increased gut permeability, consistent with the “leaky gut” hypothesis described in HF [24]. Engagement of Toll-like receptor 4 (TLR4) triggers downstream signalling cascades that activate NF-κB (Nuclear Factor kappa-light-chain-enhancer of activated B cells) and the NLRP3 (NOD-like receptor protein 3) inflammasome, resulting in the production of pro-inflammatory cytokines such as Tumour necrosis factor-α (TNF-α), Interleukin-1 beta (IL-1β), and Interleukin-6 (IL-6). Notably, while transient TLR4 activation may confer cardioprotection, sustained signalling becomes maladaptive, driving cytokine amplification and adverse cardiac remodelling. TNF-α illustrates this biphasic nature. Elevated levels have long been associated with HF and correlate with disease severity; however, their effects are highly dependent on concentration, duration of exposure, and receptor subtype. Excessive activation of Tumour Necrosis Factor Receptor 1 (TNFR1) promotes contractile dysfunction, hypertrophy, and fibrosis, whereas lower levels and TNFR2 signalling may exert protective effects. This dual aspect likely explains the failure—and possible harm—observed in clinical trials targeting TNF-α [25].

The NLRP3 inflammasome acts as a central sensor of cellular stress and an amplifier of inflammation by activating caspase-1 and promoting the maturation of IL-1β and Interleukin-18 (IL-18). In HFpEF, increased NLRP3 and IL-1β levels correlate with diastolic dysfunction, and preclinical inhibition of this pathway in animal models improves exercise capacity and diastolic function while reducing fibrosis. Cardiac macrophages add further to this complexity: resident C-C Motif Chemokine Receptor 2 (CCR2) negative macrophages are generally involved in tissue repair, whereas recruited CCR2-positive monocytes drive inflammatory responses and adverse remodelling [26,27,28].

Myocardial insulin resistance represents a key mechanistic link between metabolic dysfunction and immune activation in HF. Impaired cardiomyocyte insulin signalling contributes to altered substrate utilisation, reduced glucose uptake, mitochondrial dysfunction, and loss of metabolic flexibility, thereby promoting inflammatory activation and maladaptive remodelling [19]. Ectopic lipid accumulation, mitochondrial dysfunction, and oxidative stress activate serine kinases that impair insulin signalling and enhance NF-κB-mediated inflammatory pathways, consequently reducing glucose uptake and compromising metabolic flexibility. In conjunction with RAAS activation, endothelial dysfunction, and systemic inflammation, these processes culminate in interstitial fibrosis and diastolic dysfunction, hallmarks of HFpEF [29]. The limited success of broad anti-inflammatory therapies suggests a partial understanding of inflammatory networks in HF. However, emerging evidence suggests that targeted modulation of specific pathways in selected patient subsets may be effective. For instance, analyses from the CANTOS trial indicate that IL-1β inhibition with canakinumab reduces HF hospitalisations in patients with prior myocardial infarction and elevated inflammatory burden. In HFpEF, incretin-based therapies such as semaglutide and tirzepatide have exhibited consistent reductions in systemic inflammation, along with improvements in clinical status, while ongoing trials are evaluating IL-6 inhibition. Collectively, these data support the concept that inflammation in HF is initially adaptive and reparative but becomes maladaptive when persistent and excessive, demonstrating the need for precision-based rather than global anti-inflammatory strategies [30].

**Figure 2:**
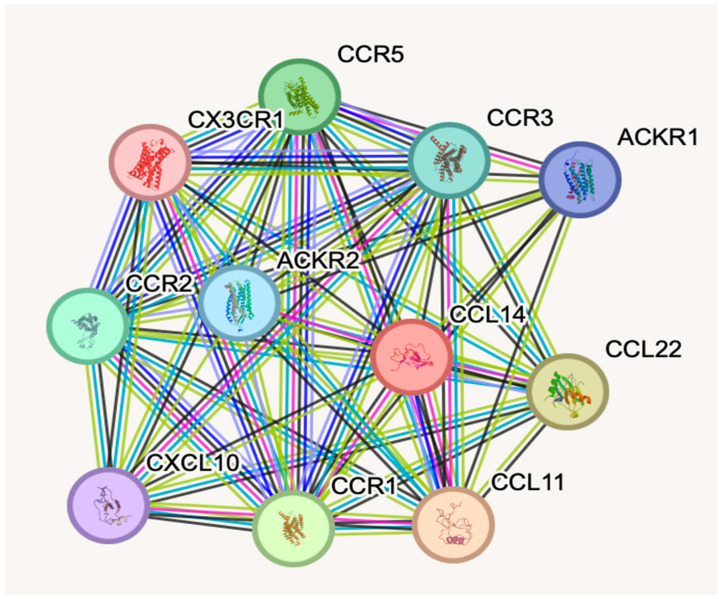
Protein-interaction network—derived from STRING database—of chemokine receptors and their ligands, illustrating the complex connectivity underlying immune signalling pathways and chemokine-mediated cellular communication. Nodes represent proteins, while coloured edges indicate different types of evidence supporting the predicted functional associations: curated databases (light blue), experimentally determined interactions (magenta), gene neighbourhood (green), gene fusion (red), gene co-occurrence (dark blue), text mining (yellow), co-expression (black), and protein homology (purple). Edge thickness reflects the confidence score of the interaction. (CCR5: C-C chemokine receptor type 5; CCR3: C-C chemokine receptor type 3; ACKR1: Atypical Chemokine Receptor 1; CX3CR1: C-X3-C Motif Chemokine Receptor 1; CCR2: C-C chemokine receptor type 2; ACKR2: Atypical Chemokine Receptor 2; CCL14: Chemokine C-C Motif Ligand 14; CCL22: Chemokine C-C Motif Ligand 22; CXCL10: C-X-C motif chemokine ligand 10; CCR1: C-C chemokine receptor type 1; CCL11: Chemokine C-C Motif Ligand 11).

In HF, the chemokine–receptor network represents a central molecular axis driving chronic inflammation and adverse cardiac remodelling. Several different stimuli, including ischaemia, mechanical stress, and neurohormonal activation, induce the expression of multiple chemokines (e.g., CCL11, CCL14, CCL22, CXCL10), which operate within a highly redundant signalling system. This functional redundancy—where ligands interact with multiple receptors and vice versa—guarantees continued leukocyte recruitment in spite of partial pathway inhibition. Chemokine receptors such as CCR1, CCR2, CCR3, CCR5, and CX3CR1 mediate the infiltration of monocytes, macrophages, and lymphocytes into the myocardium, promoting a transition from acute to chronic inflammation. These immune cells, in turn, release cytokines and growth factors that activate fibroblasts, leading to extracellular matrix deposition, fibrosis, and progressive ventricular dysfunction. Concurrently, chemokine-mediated endothelial dysfunction and microvascular impairment exacerbate tissue hypoxia, additionally amplifying myocardial injury. Although atypical chemokine receptors (e.g., ACKR1, ACKR2) help to regulate chemokine availability, their buffering capacity is often overwhelmed in HF. This intrinsic complexity and redundancy likely underlie the limited success of therapies targeting single chemokine pathways, emphasising the need for combined, system-level anti-inflammatory strategies (Figure 2) [31,32,33,34,35,36].

Endothelial dysfunction (ED) represents a central pathophysiological nexus in cardiovascular disease and plays a central role in the development and progression of HF, particularly HF with HFpEF. Driven primarily by chronic inflammation and oxidative stress, ED exerts direct deleterious effects on the vascular wall while increasing the impact of cardiometabolic comorbidities such as obesity, diabetes, and hypertension. Within the myocardium, microvascular dysfunction impairs LV relaxation, promotes maladaptive hypertrophic remodelling, and contributes to the evolution of HFpEF. In chronic HF, ED is consistently associated with adverse outcomes, irrespective of aetiology or disease severity. Impaired endothelial-dependent vasodilation leads to reduced peripheral and coronary perfusion, coupled with enhanced vasoconstriction, thereby exacerbating tissue hypoxia and further activating maladaptive neurohumoral and renal responses. At the molecular level, oxidative stress is a key driver of ED, largely through endothelial nitric oxide synthase (eNOS) uncoupling and a consequent reduction in nitric oxide (NO) bioavailability. Under pathological conditions, depletion of the essential cofactor tetrahydrobiopterin (BH_4_) shifts eNOS activity from NO production to superoxide generation, further amplifying oxidative stress. Neurohormonal activation, inflammatory mediators, and altered shear stress worsen this process, resulting in cytokine release, further eNOS dysregulation, and a progressive decline in NO signalling. The imbalance between NO and endothelin-1 (ET-1) represents a unifying mechanism linking endothelial dysfunction to impaired ventricular performance and adverse clinical outcomes. The paradigm proposed by Paulus and Tschöpe brings out the central role of coronary microvascular inflammation in the pathogenesis of HFpEF. According to this model, cardiometabolic comorbidities induce a proinflammatory state that reduces NO bioavailability and downstream cyclic guanosine monophosphate (cGMP)–protein kinase G (PKG) signalling in cardiomyocytes, producing increased myocardial stiffness, hypertrophy, and fibrosis. Coronary microvascular dysfunction (CMD), present in a substantial proportion of HFpEF patients, is associated with elevated natriuretic peptides, markers of endothelial injury, and worse right ventricular function. Chronic CMD may also promote vascular rarefaction and fibrosis, additionally impairing myocardial perfusion and function. More recently, this scheme has been expanded to include the concept of metabo-inflammation, in which nutrient excess and obesity cause sustained microvascular inflammation, nitrosative stress, and metabolic inflexibility, further disrupting NO–cGMP signalling and cellular balance [37,38,39].

ED additionally contributes directly to myocardial fibrosis through endothelial-to-mesenchymal transition (EndMT), a process in which endothelial cells acquire a fibroblast-like phenotype. This transition is regulated by oxidative stress and inflammatory signalling, with NADPH (reduced nicotinamide adenine dinucleotide phosphate) oxidase-mediated pathways playing a central role, and is further modulated by epigenetic mechanisms, including DNA methylation, histone modifications, and noncoding RNAs [40,41]. From a therapeutic perspective, ED represents a modifiable target. Indeed, the endothelial quality index (EQI), assessed by digital thermal monitoring, has recently appeared as a promising non-invasive prognostic biomarker in HF. In a study by Charfferdine et al., approximately 42.5% of patients with HFrEF exhibited baseline endothelial dysfunction. Notably, an improvement in endothelial function—defined as a ΔEQI ≥ 0.2 following 3 months of optimised medical therapy—was strongly associated with a reduction in 1-year mortality (AUC = 0.82) and rehospitalisation risk (AUC = 0.837). These findings reveal the potential utility of dynamic endothelial function assessment not only as a marker of vascular health but also as an indicator of therapeutic response and clinical prognosis in HF [42].

Lifestyle interventions, such as exercise, improve endothelium-dependent vasodilation and NO bioavailability, while established HF therapies, including ACE inhibitors, mineralocorticoid receptor antagonists (MRA), and third-generation β-blockers, exert beneficial effects on endothelial function through anti-inflammatory and antioxidant mechanisms. Statins further enhance vascular function by improving the coronary flow reserve. Emerging therapeutic strategies intend to restore NO–cGMP signalling and reduce oxidative stress, including phosphodiesterase-5 (PDE5) inhibitors, soluble guanylate cyclase (sGC) modulators, Sodium-Glucose Co-Transporter 2 (SGLT2) inhibitors, and targeted anti-inflammatory approaches [43].

### 2.3. Mitochondrial Dysfunction and Energetic Remodelling

ED and microvascular disease directly contribute to cardiomyocyte dysfunction by impairing coronary perfusion, decreasing oxygen delivery, and promoting a pro-oxidative and pro-inflammatory myocardial environment. This chronic state of metabolic and redox imbalance impairs cellular homeostasis, alters mitochondrial function, and compromises energy production. As a result, cardiomyocytes become more susceptible to injury and activate regulated cell death pathways, thereby representing a key mechanistic link in the progression of myocardial damage and HF. Cardiomyocyte dysfunction and regulated cell death pathways are critical determinants of HF pathogenesis, with distinct mechanisms operating across the ejection fraction spectrum. Given the limited capacity for cardiomyocyte renewal, their loss through apoptosis, necroptosis, autophagy-dependent cell death, ferroptosis, and pyroptosis directly impairs contractile function and drives maladaptive ventricular remodelling [44,45].

In HFpEF, free fatty acid accumulation creates myocardial fuel overload, leading to mitochondrial dysfunction and profound alterations in energetics. The heart progressively loses its capacity to adapt substrate utilisation in response to nutritional status, energy substrate availability, and haemodynamic load, resulting in inefficient ATP generation and adverse remodelling. HFpEF hearts exhibit decreased phosphocreatine/ATP ratio and high fatty acid oxidation dependency, yet demonstrate blunted mitophagy despite metabolic stress, suggesting impaired mitochondrial quality control. This maladaptation is accompanied by reduced respiratory function, increased reactive oxygen species production, NAD^+^ depletion, and hyperacetylation of oxidative enzymes. Adipocyte-derived factors directly impair cardiomyocyte contractility by reducing intracellular calcium transients, while toxic metabolic intermediates trigger cardiomyocyte death and fibrotic remodelling, collectively disrupting energetics, blunting contractile reserve, and promoting ventricular stiffening and diastolic dysfunction [46,47].

In contrast, HFrEF is characterised by direct cardiomyocyte death driven by oxidative stress from ischaemia, infection, or toxic agents, triggering exaggerated autophagy, apoptosis, and necrosis, resulting in replacement fibrosis with patchy collagen deposition, left ventricular dilation, and eccentric remodelling. Network analysis of 92 biomarkers reveals that pathways unique to HFrEF involve DNA-binding transcription factor activity, cellular protein metabolism, and regulation of nitric oxide biosynthesis, whereas HFpEF pathways involve cytokine response, extracellular matrix organisation, inflammation, neutrophil degranulation, and integrin signalling, emphasising the need for phenotype-specific therapeutic approaches [48].

### 2.4. Regulated Cell Death and Maladaptive Remodelling

Apoptosis operates through extrinsic (death receptor) and intrinsic (mitochondrial) pathways, while necroptosis, mediated by the RIPK1-RIPK3-MLKL (Receptor-interacting serine/threonine-protein kinase 1–Receptor-interacting serine/threonine-protein kinase 3–Mixed Lineage Kinase Domain-Like pseudokinase) axis, promotes inflammation and fibrotic remodelling [49,50,51].

Pharmacological RIPK3 inhibition with GSK′872 suppresses necroptosis, normalises dysregulated miRNAs, decreases the collagen content, and mitigates inflammation, though effects vary by aetiology. Autophagy, although generally cytoprotective, becomes maladaptive when dysregulated, yet promoting autophagy through caloric restriction, resveratrol, or exercise reduces infarct size and induces reverse remodelling, suggesting dual treatment interventions: anti-apoptosis in noncardiomyocytes to preserve wall thickness and pro-autophagy in cardiomyocytes to compensate for energy insufficiency [52].

Moreover, ferroptosis, an iron-dependent form of regulated cell death driven by lipid peroxidation, has emerged as one of the most promising targets for treatment in HF. Ferroptosis is abundant across multiple HF models (ischaemic, pressure overload, diabetic, septic, obesity-related, doxorubicin-induced), where disordered iron handling, Glutathione peroxidase 4 (GPX4) degradation, glutathione depletion, phospholipid peroxidation, and mitochondrial stress converge to cause contractile dysfunction and adverse remodelling. The “ferroptosis nexus” framework proposes that iron mobilisation (Nrf2-mediated HO-1 upregulation), antioxidant collapse (GPX4 inactivation), lipid priming (accumulation of polyunsaturated fatty acid-containing phospholipids), and mitochondrial/calcium amplifiers form a self-reinforcing loop culminating in pump failure. Blockade of ferroptosis with ferrostatin-1, liproxstatin-1, or iron chelators rescues systolic/diastolic indices and reverses remodelling, while cardiometabolic drugs with clinical efficacy—SGLT2 inhibitors, sacubitril/valsartan, finerenone, levosimendan, and nicorandil—restore function partly through ferroptosis inhibition. Humans with failing myocardium and epicardial adipose tissue exhibit ferroptosis-specific transcriptional and lipidomic signatures, while patients receiving SGLT2 inhibitors show reduced ferroptosis activity, providing translational evidence for therapeutic targeting [53,54,55,56,57].

Pyroptosis, a pro-inflammatory form of cell death mediated by gasdermin proteins through canonical (caspase-1) and non-canonical (caspase-11/4/5) inflammasome pathways, plays a major role in HF pathogenesis. The NLRP3 inflammasome senses myocardial injury and triggers caspase-1 activation, cleavage of pro-IL-1β and pro-IL-18, and pyroptotic cell death. Haemodynamic stress induces sterile inflammation through wall tension and mechanical stretch, triggering proinflammatory cytokine release, mitochondrial dysfunction, reactive oxygen species generation, and NLRP3 activation. In dilated cardiomyopathy, hyperactivated NLRP3 inflammasome with pyroptotic death negatively correlates with cardiac function, mechanistically driven by NADPH Oxidase 1/NADPH Oxidase 4 (NOX1/NOX4) expression and Dynamin-related protein 1 (Drp1)-mediated mitochondrial fission. Blocking IL-1β with canakinumab reduced hospitalisations and mortality in HF patients, though pyroptosis operates predominantly in post-MI HF but not pressure overload-induced HF, highlighting aetiology-specific mechanisms [26,30,58,59,60,61].

The detailed interplay between regulated cell death pathways amplifies myocardial damage, with TLR4/NOX4 signalling promoting both autophagy and ferroptosis, and RIPK3 serving as a convergent point for necroptosis and pyroptosis, suggesting that multi-targeted approaches may display superior cardioprotection to single-pathway inhibition. Therapeutic methods must be phenotype-tailored: in HFrEF, direct inhibition of apoptosis, necroptosis, and ferroptosis may be beneficial, whereas in HFpEF, interventions targeting mitochondrial quality control (enhancing fatty acid oxidation to stimulate mitophagy), NAD^+^ precursor supplementation, mTORC1 (mammalian target of rapamycin complex 1) inhibition, and β-hydroxybutyrate administration show promise. SGLT2 inhibitors and Glucagon-Like Peptide-1 (GLP-1) receptor agonists confer clinically proven benefits in HFpEF via systemic metabolic reprogramming toward oxygen-efficient substrates, attenuation of inflammation, and reduction in ferroptosis. Standardised ferroptosis signatures, single-cell and spatial transcriptomics analysis, and mechanism-driven clinical trials are needed to identify responsive HF phenotypes and translate regulated cell death modulation into precision cardioprotection [62].

**Figure 3:**
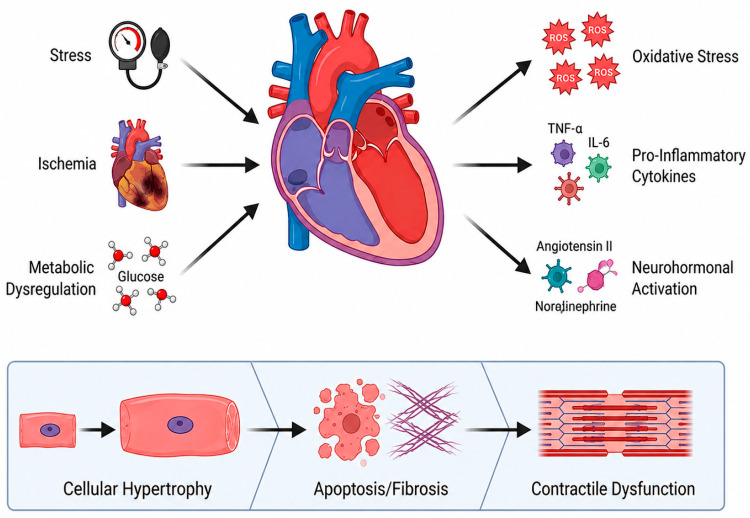
Integrated overview of molecular and cellular mechanisms driving HF progression. Abbreviations: ROS, reactive oxygen species; TNF-α, tumour necrosis factor-alpha; IL-6, interleukin-6.

In HF, neurohormonal activation, chronic inflammation, ED, and regulated cell death are tightly interconnected processes that drive disease progression at multiple levels (Figure 3). This complex network of signals converges on the regulation of gene expression, triggering adaptive and maladaptive responses that go beyond transient molecular changes. Increasing evidence suggests that such stimuli can induce stable modifications in chromatin structure and gene activity, thereby shaping long-term cellular behaviour. In this context, epigenetic remodelling appears to be a critical mechanism linking upstream pathological triggers to persistent alterations in cardiac phenotype, thereby creating the conditions for the processes discussed in the following section.

## 3. Genomic, Epigenetic, and Transcriptomic Regulation

The genetic architecture of HF spans the full spectrum of allelic frequencies. Both common and rare variants distinctly contribute to disease susceptibility and progression. Recent genome-wide association studies (GWAS) have identified 176 genome-wide significant risk loci in over 2.3 million individuals. These loci group into five main functional modules: anthropometric traits/obesity, blood pressure/renal function, atherosclerosis/lipid metabolism, immune activity, and arrhythmias. In parallel, exome sequencing analyses of 376,334 individuals show significant associations with rare loss-of-function variants in Mendelian cardiomyopathy genes, especially Titin (*TTN*), Myosin Binding Protein C3 (*MYBPC3*), Filamin C (*FLNC*), and BCL2-Associated Athanogene 3 (*BAG3*). This dichotomy emphasises a key point: heritability due to rare coding variants is highly concentrated within a few cardiomyopathy genes, while that of common variants spreads diffusely across the genome [63].

Gene prioritisation analyses using colocalization approaches and transcriptome-wide association studies have identified both established and previously unreported candidate genes. These candidates have been confirmed through gene expression profiling in failing and non-failing human hearts. Convergent evidence supports a role for 2-oxoisovalerate dehydrogenase subunit alpha (*BCKDHA*) and circulating branched-chain amino acids in cardiac structure and function, as demonstrated by Mendelian randomisation. Proteome-wide Mendelian randomisation studies have also identified seven proteins as possible therapeutic targets for the primary prevention of HF: Calcium/calmodulin-dependent protein kinase type II delta chain (CAMK2D), Serine/threonine-protein kinase D1 (PRKD1), PRKD3, Mitogen-activated protein kinase 3 (MAPK3), TNF-related weak inducer of apoptosis (TNFSF12), Apolipoprotein C-III (APOC3), and NEDD8 Activating Enzyme E1 Subunit (NAE1) [64,65].

A key observation emerging from recent studies is that the common polygenic background modifies HF risk among carriers of pathogenic truncating variants in *TTN*, suggesting that the penetrance of Mendelian variants is not exclusively determined by the monogenic genotype but is influenced by the overall polygenic burden. This finding illustrates the polygenic component of HF that is not captured by current clinical genetic testing. Carriers of pathogenic or likely pathogenic variants in genes associated with inherited cardiomyopathies exhibit a 70% increased risk of mortality and more than a twofold higher risk of HF and atrial fibrillation, despite substantial overlap in left ventricular ejection fraction with the general population [66].

Polygenic risk scores (PRS) for HF, derived from over one million single-nucleotide variants, have shown strong predictive performance among the cardiovascular risk spectrum. Individuals with intermediate and high PRS show approximately twofold and fivefold increased risk of incident HF, respectively, after adjustment for clinical risk factors, with the area under the Receiver Operating Characteristic (ROC) curve improving from 0.787 to 0.822 upon inclusion of PRS in clinical models. These data have been replicated in low-cardiovascular-risk populations with up to two decades of follow-up. Recent cross-ancestry meta-analyses in the Japanese population have identified 19 novel loci, including a common non-coding variant in *TTN* (rs1484116) associated with reduced cardiac function and worse long-term mortality. The generated PRS precisely identified individuals at risk of early-onset HF and increased mortality [67,68].

Pharmacogenomic variants represent an additional dimension of precision medicine in HF. The *ADRB1*Arg389Gly polymorphism has shown that patients who are homozygous for Arg389 experience a 38% reduction in mortality with bucindolol therapy, whereas Gly389 carriers derive no significant benefit. The G Protein-Coupled Receptor Kinase 5G (*GRK5*) Leu41 variant, with approximately a 10-fold higher allele frequency in individuals of African ancestry (0.23 vs. 0.02), is associated with a lack of response to β-blocker therapy, suggesting an intrinsic β-blocking effect. In the A-HeFT trial, the G protein subunit beta 3 (*GNB3*) Thr825 variant predicted an enhanced therapeutic response to hydralazine–isosorbide dinitrate, with markedly improved event-free survival among Thr825 homozygotes. Polymorphisms in Cytochrome P450 2D6 (*CYP2D6*) influence the pharmacokinetics of metoprolol and carvedilol, with poor metabolisers exhibiting heart rate and blood pressure reductions of 8.5 bpm and 5 mmHg, respectively, compared to extensive metabolisers at equivalent doses, and an approximately fourfold increased risk of bradycardia [69].

Combining PRS with clinical risk factors, lifestyle variables, and social determinants of health results in improved predictive performance compared to individual risk scores, with an AUC of 0.763. This highlights the value of integrating multiple domains for HF prediction.

Ongoing challenges remain for clinical implementation. These include substantial inter-individual variability, the need for standardised reporting, and the immediate need to increase diversity in PRS studies. Enhancing diversity in PRS studies could improve predictive accuracy for historically underrepresented populations [70,71]. Genetic susceptibility and disease-modifying variants define the risk landscape, although they do not fully explain phenotypic variability. Epigenetic mechanisms—especially DNA methylation along with histone modifications—are dynamic regulators. They link genetic predisposition, environment, and transcriptional remodelling in HF.

DNA methylation orchestrates the metabolic switch that characterises HF, in which promoter hypermethylation suppresses oxidative metabolism genes, while hypomethylation activates glycolytic pathways [72,73]. Recent genome-wide analyses (2025) have identified 56 CpG loci associated with HF phenotypes, including 18 loci that are predictive of disease progression (e.g., 5′-AMP-activated protein kinase subunit gamma-2 -*PRKAG2*-, ankyrin Repeat and Sterile Alpha Motif Domain Containing 1A—*ANKS1A*- and Motile Sperm Domain Containing 3—*MOSPD3*). Notably, the DNA methyltransferase inhibitor RG108 has been shown to attenuate cardiac hypertrophy and preserve ejection fraction, underscoring the therapeutic potential of epigenetic targeting [74]. However, LV assist device support normalises only 3.2% of differentially methylated sites, suggesting that epigenetic alterations are largely resistant to reversal by mechanical unloading [75]. The transcriptomic configuration of the failing heart is governed by druggable chromatin checkpoints, particularly the balance between histone deacetylases (HDACs) and histone acetyltransferases (p300/CBP), as well as acetyl-lysine “reader” proteins such as the Bromodomain and Extra-Terminal domain (BET) family, including Bromodomain-containing protein 4 (BRD4), which amplify hypertrophic and profibrotic gene programmes. The acetyltransferases p300/CBP are both necessary and sufficient to drive pathological hypertrophy through acetylation of transcription factors such as Myocyte Enhancer Factor 2 (MEF2) and transcription factor GATA-4, whereas sirtuins (SIRT1, SIRT6, SIRT7) exert cardioprotective effects via NAD^+^-dependent mechanisms [76].

Pharmacological targeting of these pathways has shown translational promise. Givinostat, an HDAC inhibitor, improves diastolic dysfunction with preserved ejection fraction in mouse models through a non-genomic mechanism that improves myofibrillar relaxation. Similarly, the BET inhibitor apabetalone, evaluated in the BETonMACE trial, has been associated with reduced HF hospitalisation in patients with diabetes and acute coronary syndrome [77,78].

The Inositol requiring 80 (INO80) chromatin remodelling complex has been established as a particularly compelling therapeutic target: its overexpression induces rapid-onset HF within days through reprogramming of MEF2-dependent transcriptional networks, whereas conditional deletion in established disease models markedly preserves cardiac function [79]. Beyond linear chromatin modifications, reorganisation of three-dimensional chromatin topology contributes to transcriptional reprogramming, with extensive rewiring of acetylation of lysine 27 on histone H3 protein subunit (H3K27ac)-associated enhancer loops observed in dilated cardiomyopathy. These regions are enriched for Heart- And Neural crest Derivatives-expressed protein 1 (HAND1), a key regulator of early cardiogenesis whose reactivation drives maladaptive dilative remodelling [80].

An emerging conceptual framework highlights the bidirectional relationship between metabolism and epigenetics, whereby metabolic intermediates such as acetyl-CoA, NAD^+^, and α-ketoglutarate shape the epigenetic landscape, while epigenetic dysregulation reciprocally suppresses oxidative phosphorylation pathways [81]. Collectively, these data position epigenetics as a mechanistic substrate explaining the persistence of cardiac injury beyond its initial trigger, and as a rational therapeutic entry point through targeting chromatin readers, writer–eraser balance, and enhancer regulation. A central translational priority continues the identification of causal, cell-type- and stage-specific regulatory nodes to support precise and effective intervention.

A hallmark of heart failure (HF) is the reciprocal relationship between cellular metabolism and epigenetic regulation. Beyond serving as energetic substrates, metabolic intermediates directly modulate chromatin-modifying enzymes, thereby influencing transcriptional programmes related to hypertrophy, inflammation, fibrosis, mitochondrial adaptation, and cardiomyocyte survival. Consequently, the metabolic state of the failing myocardium acts as a dynamic epigenetic regulator.

Multiple metabolites serve as essential cofactors or substrates for epigenetic enzymes. Acetyl CoA, generated via glucose oxidation, fatty acid oxidation, and citrate metabolism, is the principal acetyl donor for histone acetyltransferases (HATs) such as p300/CBP. Increased acetyl CoA concentrations enhance histone acetylation and promote transcription of genes associated with hypertrophic growth and metabolic remodelling. Conversely, impaired mitochondrial oxidative metabolism and diminished acetyl CoA flux can decrease chromatin accessibility and suppress oxidative phosphorylation.

NAD^+^ functions as a critical metabolic sensor linking mitochondrial activity to chromatin regulation through activation of sirtuins, particularly SIRT1, SIRT3, and SIRT6. These NAD^+^-dependent deacetylases regulate histone acetylation, mitochondrial biogenesis, oxidative stress responses, and inflammatory signalling [81]. In HF, depletion of NAD^+^ pools resulting from mitochondrial dysfunction and oxidative stress reduces sirtuin activity, leading to hyperacetylation of histones and metabolic enzymes, impaired mitochondrial respiration, and activation of pro-inflammatory and profibrotic transcriptional programmes.

Alpha-ketoglutarate, a central intermediate of the tricarboxylic acid cycle, serves as an essential cofactor for Ten-Eleven Translocation (TET) DNA demethylases and Jumonji C-domain demethylases. Reduced alpha-ketoglutarate availability during mitochondrial dysfunction can impair DNA and histone demethylation, thereby stabilising maladaptive epigenetic states. In contrast, accumulation of metabolites such as succinate and fumarate may inhibit alpha-ketoglutarate-dependent dioxygenases, resulting in sustained inflammatory and hypoxic transcriptional responses.

Additional metabolic signals contribute to epigenetic regulation. S-adenosylmethionine (SAM), generated by one-carbon metabolism, acts as the universal methyl donor for DNA and histone methyltransferases. Altered redox balance influences epigenetic signalling through reactive oxygen species (ROS)-mediated modulation of chromatin remodelling complexes and DNA methylation patterns. Furthermore, beta-hydroxybutyrate, which is increasingly utilised in advanced HF, may function as an endogenous histone deacetylase inhibitor, thereby linking ketone metabolism to transcriptional adaptation [62].

In summary, metabolic remodelling in HF is not solely a consequence of energetic dysfunction but also acts as a driver of stable epigenetic reprogramming. The metabolic–epigenetic axis likely maintains maladaptive transcriptional states even after the initial pathological trigger has been eliminated. Elucidating these interactions may facilitate the development of precision therapies that target metabolic pathways and chromatin-regulating enzymes in a coordinated manner.

While DNA methylation and histone modifications define the chromatin architecture of the failing heart, they operate in concert with additional layers of post-transcriptional regulation. Regulatory non-coding RNAs, including microRNAs (miRNAs), long non-coding RNAs (lncRNAs), and circular RNAs (circRNAs), fine-tune gene expression networks, acting as critical intermediaries that integrate epigenetic signals into coordinated transcriptional and translational responses. Non-coding RNAs (ncRNAs) orchestrate post-transcriptional regulation in HF, modulating key processes such as hypertrophy, fibrosis, β-adrenergic signalling, and calcium homeostasis. Meta-analyses have identified a core signature of 16 differentially expressed microRNAs, including upregulation of miR-21, miR-214, and miR-27b, and downregulation of miR-133a, miR-29a/b, miR-1, and miR-150. Among these, miR-133 and miR-1 regulate adrenergic responsiveness, while miR-208 controls the balance between α- and β-myosin heavy chain isoforms [82,83].

Circulating microRNA profiles discriminate HF with reduced versus preserved ejection fraction (HFrEF vs. HFpEF) and predict the response to cardiac resynchronisation therapy, with *miR-30d* outdoing conventional clinical parameters such as QRS duration [84].

Therapeutically, antagomir-based strategies have shown notable promise: inhibition of *miR-25* restores sarcoplasmic/endoplasmic reticulum Ca2+ ATPase 2a (SERCA2a) activity, improving LV function and survival, whereas targeting *miR-208a* prevents adverse remodelling in hypertensive models [85]. More broadly, noncoding RNAs are increasingly recognised as key regulatory elements in cardiovascular disease, integrating diagnostic, mechanistic, and therapeutic dimensions in heart failure [86]. lncRNAs additionally contribute to epigenetic regulation. Myosin Heavy chain-associated RNA Transcript (*Mhrt*) exerts cardioprotective effects through interaction with the chromatin remodeler Brahma-related gene 1 (BRG1), Cardiac Hypertrophy-Associated Transcript (*Chast*) promotes hypertrophy by inhibiting autophagy, and Cardiac Hypertrophy-Associated Epigenetic Regulator (*Chaer*) modulates epigenetic reprogramming via interaction with Polycomb Repressor Complex 2 (PRC2). Silencing of these lncRNAs attenuates pathological remodelling, while circulating Long Intergenic non-coding RNA Predicting CARdiac remodelling (*LIPCAR*) levels have been shown to predict three-year cardiovascular mortality [87].

CircRNAs, characterised by their covalently closed structure, present remarkable persistence and resistance to degradation. Acting primarily as microRNA “sponges,” they are emerging as promising biomarkers, especially because of their stability within exosomes [87]. The combination of multi-marker non-coding RNA (ncRNA) panels, together with emerging therapeutic approaches, including antisense oligonucleotides, Clustered Regularly Interspaced Short Palindromic Repeats/dead CRISPR-associated protein 9 (CRISPR/dCas9)-based epigenome editing, and advanced delivery systems such as exosomes and nanoparticles, has the potential to transform risk stratification and enable truly personalised therapeutic strategies in HF (Table 1) [88,89].

## 4. Proteomics and Post-Translational Modifications

**Figure 4:**
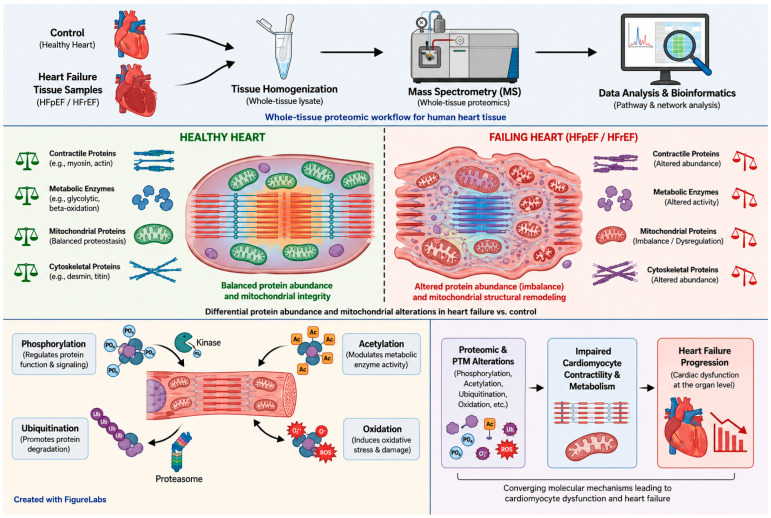
Proteomic alterations and post-translational regulation in heart failure (PTM: Post-translational modifications). Green indicates balanced/physiological protein abundance in the healthy heart, whereas red indicates altered protein abundance and proteomic imbalance in the failing heart. Scale icons denote protein balance or imbalance. Abbreviations: MS, mass spectrometry; PTM, post-translational modification; ROS, reactive oxygen species; Ac, acetylation; Ub, ubiquitination; PO_4_, phosphorylation.

HF is a complex syndrome characterised by marked molecular alterations that vary according to aetiology and left ventricular ejection fraction. Advances using high-resolution proteomic technologies have enabled the identification of myocardial molecular signatures, revealing hundreds of differentially expressed proteins compared to healthy controls and demonstrating that HFrEF, HFmrEF, and HFpEF represent biologically distinct entities. Proteomic analyses have consistently revealed three major functional categories: energy metabolism, cytoskeletal organisation, and stress response, with marked downregulation of oxidative phosphorylation, substrate metabolism, and protein translation proteins, alongside upregulation of inflammatory, immune, and oxidative stress-related proteins, indicating the myocardium’s maladaptive response to chronic haemodynamic stress (Figure 4). Aetiology-specific signatures have also been described: ischaemic cardiomyopathy shows selective upregulation of Ubiquinol-Cytochrome c Reductase Complex III Subunit VII (UQCRQ), Glucose Transporter Type 4 (GLUT4), and adiponectin; HFrEF is associated with the activation of growth-related pathways, including growth differentiation factor-15; and HFpEF is characterised by increased inflammation and pronounced metabolic alterations, particularly in patients with severe obesity [90,91].

Notably, HFpEF exhibits transcriptome–proteome discordance, in which oxidative metabolism genes are transcriptionally upregulated, yet their corresponding proteins are reduced, denoting a primary defect in protein translation and defining distinct patient subgroups with differential proteomic profiles. Phosphoproteomic analyses add further complexity, identifying 33 differentially phosphorylated proteins in post-infarction HF, enriched in nucleocytoplasmic transport and mRNA surveillance pathways, with phosphorylated BCL2-associated transcription factor 1 (Bclaf1) Ser658 emerging as a potential regulator of cardiomyocyte apoptosis and upstream kinases, including AMP-activated Protein Kinase (AMPK), Protein Kinase A (PKA), and p21-Activated Kinase 1 (PAK1), representing candidate treatment targets. Overall, myocardial proteomic signatures provide mechanistic insight into HF heterogeneity, offer options for precision clinical interventions targeting energy metabolism and protein translation, and identify plasma biomarkers, such as angiopoietin-2 and thrombospondin-2, with potential utility for prompt diagnosis and disease monitoring [92].

Proteomic profiling of the failing heart highlights altered proteins, yet functional outcomes are further determined by post-translational modifications such as phosphorylation, acetylation, and redox signalling, which dynamically regulate protein activity and stability, linking molecular signatures to cardiac dysfunction. Post-translational modifications (PTMs) are fundamental regulatory mechanisms in cardiac pathophysiology, modulating protein function rapidly without requiring new protein synthesis. Among the most relevant PTMs in HF, phosphorylation, acetylation, and redox-based modifications act as central regulators of contractile function, energy metabolism, and stress responses, and constitute promising therapeutic objectives. In HFpEF, widespread hyperphosphorylation is a dominant feature, with 98% of 529 significantly altered phosphorylation sites increased, relative to controls, and 39% localised to the sarcomeric Z-disc. PKC (Protein Kinase C) isoforms play key roles: PKCα induces hyperphosphorylation of myofilament proteins in end-stage HF, reducing maximal force by 30% and calcium sensitivity by 55%, while PKCβ appears as the dominant kinase in HFpEF, and interventions such as cardiac stem cell therapy that selectively reverse PKCβ upregulation partially normalise hyperphosphorylation and restore diastolic function [93]. PKC-mediated phosphorylation of cardiac troponin I at S44 contributes to cellular contractile dysfunction in both human HF and animal models, and PKCα inhibition reduces cardiac Troponin I phosphorylated at Serine 44 (cTnI p-S44), improving function [94]. Calcium cycling proteins are critical targets: Ryanodine Receptor type 2 (RyR2), when hyperphosphorylated by PKA or Calcium/Calmodulin-dependent protein Kinase II (CaMKII) and simultaneously oxidised and nitrosylated, becomes “leaky,” causing diastolic calcium loss, depletion of sarcoplasmic calcium stores, impaired systolic contraction, and fatal arrhythmias, while hypophosphorylation of phospholamban reduces SERCA2a activity, additionally impairing calcium reuptake [95]. Lysine acetylation profoundly regulates mitochondrial metabolism, with over 60% of mitochondrial proteins acetylated; in HFpEF, global mitochondrial hyperacetylation predominates, with 74% of hyperacetylated proteins enriched in the Krebs cycle, oxidative phosphorylation, and fatty acid oxidation, associated with reduced NAD^+^/NADH ratios, mitochondrial dysfunction, and Krebs cycle metabolite depletion [96].

Moreover, Dihydrolipoamide S-acetyltransferase (Dlat) acts as a key transacetylase, acetylating Hydroxyacyl-CoA Dehydrogenase Trifunctional Multienzyme Complex Subunit Alpha (HADHA) at K728, inactivating enzymatic activity and limiting fatty acid oxidation; Dlat overexpression exacerbates acetylation and metabolic dysfunction, whereas knockdown lessens these effects. Mitochondrial acetyltransferase Males absent On the First (MOF), upregulated in HF, acetylates ATP Synthase F1 Subunit Beta (ATP5B) K201, impairing respiration, and cardiomyocyte-specific deletion of General Control of Amino acid synthesis 5-Like 1 (GCN5L1) reduces mitochondrial acetylation, enhances fatty acid oxidation, and improves diastolic function [97,98,99].

Redox modifications additionally modulate cardiac function, as Reactive Oxygen Species (ROS), primarily mitochondrial in origin, serve as physiological signalling molecules but, at pathological levels, alter excitation–contraction coupling and calcium handling, and promote arrhythmias, hypertrophic signalling, apoptosis, and matrix remodelling. RyR2 oxidation, nitrosylation, and hyperphosphorylation exemplify the link between redox and phosphorylation in mediating sarcoplasmic calcium leak [100,101].

Therapeutically, PTMs intersect in complex networks: mitochondrial hyperacetylation can inhibit Superoxide Dismutase 2 (SOD2), amplifying oxidative stress, while ROS modulate kinase and phosphatase activities. Emerging interventions include RyR2 stabilisers, SERCA2a gene therapy, nicotinamide riboside supplementation to normalise NAD^+^/NADH and reduce acetylation, PKCβ inhibition, and mitochondria-targeted ROS modulation, highlighting PTMs as a mechanistic framework for personalised therapies customised to the specific molecular derangements underlying cardiac dysfunction.

## 5. Metabolomics and Energetic Remodelling

Mitochondrial dysfunction and bioenergetic insufficiency are fundamental contributors to the pathogenesis and progression of HF, serving as early and self-perpetuating drivers of myocardial injury. In cardiomyocytes, diminished metabolic flexibility impairs the capacity to adapt substrate metabolism to changing functional demands, resulting in reduced mitochondrial oxidation of fatty acids and glucose (Table 2). In the healthy adult heart, fatty acid β-oxidation is the primary pathway for ATP production, with glucose and other substrates contributing to a lesser extent. During HF, there is a metabolic shift away from fatty acid oxidation toward increased reliance on glucose uptake, glycolysis, and, in advanced stages, ketone body utilisation. However, mitochondrial dysfunction impairs oxidative phosphorylation, so increased glycolysis does not correspond to increased glucose oxidation. This leads to a marked reduction in ATP production and intracellular accumulation of partially oxidised metabolic intermediates, such as diacylglycerols and ceramides, which exert direct cytotoxic effects [20,102,103].

A chronic energy deficit directly leads to lipotoxicity and glucotoxicity, both of which raise oxidative stress. This, in turn, worsens mitochondrial dysfunction and activates cell death pathways, ultimately resulting in bioenergetic insufficiency. Consequently, cardiomyocytes cannot sustain the ATP-dependent processes necessary for ionic homeostasis and efficient calcium cycling. Reduced ATP impairs the performance of essential ion pumps, including Na^+^/K^+^- ATPase and sarcoplasmic reticulum Ca^2+^- ATPase. This dysfunction leads to compromised sarcoplasmic reticulum function and decreased contractility. Combined with cytosolic calcium overload, ATP depletion raises the risk of both apoptotic and necrotic cell death, intensifying cardiomyocyte loss and hastening the progression of HF.

Building on this, as HF progresses, the myocardium exhibits both quantitative and qualitative deterioration of the mitochondrial compartment. This deterioration, manifesting as reduced mitochondrial density, ultrastructural abnormalities, and disorganisation of mitochondrial cristae, leads to impaired oxidative phosphorylation. This impairment causes a shift in metabolism from predominant fatty acid and glucose oxidation to a less efficient reliance on glycolysis. Because glycolysis cannot compensate for the resultant ATP deficit, the energy deficiency worsens, further aggravating the contractile dysfunction.

Beyond metabolic impairment, mitochondrial morphological remodelling is a key hallmark of heart failure (HF) progression. In failing cardiomyocytes, increased mitochondrial fragmentation, loss of cristae integrity, matrix swelling, and disruption of the mitochondrial network architecture are observed. These structural abnormalities are closely associated with impaired oxidative phosphorylation, decreased ATP synthesis, altered calcium buffering, and heightened susceptibility to reactive oxygen species generation and cell death. Dysregulation of mitochondrial dynamics, especially excessive fission mediated by Dynamin-related protein 1 (Drp1) and diminished mitochondrial fusion, further exacerbates energetic inefficiency and maladaptive cardiac remodelling. Mitochondrial fragmentation is particularly pronounced in heart failure with preserved ejection fraction (HFpEF), where it correlates with metabolic inflexibility, oxidative stress, and impaired mitochondrial quality control.

Simultaneously, dysfunction of the mitochondrial electron transport chain leads to excessive ROS production. This phenomenon is primarily driven by electron leakage under conditions of NADH accumulation and elevated mitochondrial membrane potential, as well as by structural and functional alterations of the respiratory complexes. Specifically, when electron flow to complex IV is diminished, electrons may escape from complexes I and III, reducing molecular oxygen to superoxide rather than water. Notably, this process is further intensified by high mitochondrial membrane potential and NADH accumulation, both of which promote partial oxygen reduction and increased ROS generation [104]. Excess ROS and mitochondrial calcium overload potently trigger mitochondrial permeability transition pore opening, leading to dissipation of the inner membrane potential, collapse of oxidative phosphorylation, and matrix swelling. These mitochondrial events sequentially result in irreversible ATP loss, directly promoting necrotic cell death and inducing inflammation. Activation of apoptotic pathways in cardiomyocytes, mediated by outer mitochondrial membrane permeabilization and cytochrome c release, triggers a progressive, regulated loss of functional cells. In contrast, a severe and prolonged energy deficit leads to cellular necrosis—characterised by ATP depletion, membrane disintegration, and release of pro-inflammatory mediators—which elicits an acute inflammatory response. Both apoptotic and necrotic pathways ultimately drive pathological ventricular remodelling by promoting interstitial fibrosis, maladaptive hypertrophy, and ongoing cardiomyocyte loss.

Against this backdrop, the myocardium adapts to chronic stress and impaired contractile function through both adaptive and maladaptive changes in cardiac metabolism, shaped by hormones such as insulin, thyroid hormones, and catecholamines. In the healthy heart, fatty acid β-oxidation is the main source of ATP, and its activity is partly regulated by hormones. In HF, however, reduced mitochondrial oxidative capacity and altered hormone signalling shift the metabolism to greater glycolysis and ketone body use, with less glucose and amino acid oxidation. This shift is driven by changes in gene expression, such as downregulation of PPARα and PGC-1α, and by post-translational changes affecting key enzymes in cardiac energy metabolism.

PPARα is the main transcriptional regulator of myocardial fatty acid oxidation; its downregulation reduces the expression of β-oxidation enzymes, thereby directly promoting a shift toward glycolysis and ketone body utilisation. Similarly, downregulation of PGC-1α—a coactivator of PPARα and master regulator of mitochondrial biogenesis—further diminishes mitochondrial oxidative capacity and contributes to energetic inefficiency.

Proteins and enzymes central to cardiac metabolic pathways undergo various post-translational modifications, including phosphorylation, acetylation, and ubiquitination. These modifications are driven by hormone-mediated signalling pathways activated by insulin, catecholamines, and thyroid hormones, as well as by cellular stress signals, including activation of AMPK and PKC and changes in cellular redox status. These stress- and hormone-responsive pathways impair PPARα/PGC-1α signalling and the activity of their downstream targets, further reducing the efficiency of β-oxidation and mitochondrial respiratory chain function. Additionally, the accumulation of metabolic intermediates and mitochondrial dysfunction promotes epigenetic modifications that perpetuate the transcriptional repression of these metabolic regulators [7,105].

Metabolic remodelling in HF alters substrate utilisation, metabolite trafficking, and inter-organ metabolic communication, thereby reshaping the circulating metabolome. Changes in the metabolome, such as elevated levels of ketone bodies and acylcarnitines, increased lactate, and altered amino acid profiles (for example, decreased branched-chain amino acids, creatine, and alanine), as well as increased concentrations of citric acid cycle intermediates (such as malic acid, fumaric acid, and citric acid), serve as dynamic biomarkers for risk stratification and prognosis in HF [106]. Metabolomic panels can surpass traditional markers such as B-type natriuretic peptide (BNP) in predicting mortality and hospitalisation, while dynamic profiling, including responses to metabolic challenges, enables personalised risk assessment and guides mechanism-based therapeutic interventions. For instance, panels of metabolites, such as histidine, phenylalanine, spermidine, and phosphatidylcholine C34:4, demonstrate diagnostic accuracy comparable to BNP and superior prognostic value for predicting adverse outcomes, including death or hospitalisation. Additionally, changes in acylcarnitine profiles, particularly elevations in long-chain acylcarnitines (C16, C18:1), are associated with more severe HF and increased risk of mortality and hospitalisation; these levels decrease with mechanical circulatory support, indicating the reversibility of metabolic derangement. Alterations in ketone bodies, amino acids, and tricarboxylic acid cycle (TCA) intermediates are stage-specific: early HF may present with increased ketone bodies, whereas severe dysfunction is characterised by their depletion and accumulation of TCA intermediates. These biomarkers correlate with worsening cardiac metabolism, reflect the severity of metabolic remodelling, and are associated with disease stage, adverse outcomes, and therapeutic response [69,107].

## 6. Integrated Multi-Omics Signatures and System Biology

The integration of multi-layered data in heart failure (HF) constitutes a significant advancement in precision cardiovascular medicine. This approach systematically combines information from diverse biological and clinical domains, including genomics, transcriptomics, proteomics, metabolomics, epigenomics, advanced imaging, clinical phenotyping, and device-derived data. By transcending traditional classifications based on single metrics, such as left ventricular ejection fraction or natriuretic peptide levels, this strategy seeks to address the biological heterogeneity of HF and facilitate more nuanced mechanistic understanding and therapeutic stratification.

Multi-omic integration serves as the foundational pillar of this strategy. By simultaneously analysing genomic, transcriptomic, proteomic, and metabolomic datasets, this approach enables the identification of biological pathways involved in HF progression, the discovery of novel biomarkers, and the delineation of potential therapeutic targets. Recent studies have shown that integrated multi-omic analyses can reveal distinct molecular subtypes (endophenotypes) characterised by varying risks of adverse clinical outcomes and heterogeneous therapeutic responses, thereby supporting more targeted clinical trial designs and personalised treatment strategies. In addition, a network medicine approach facilitates integrating genetic variants, epigenetic modifications, and proteomic and metabolic profiles within a unified systems-level framework. This perspective enhances understanding of complex, multiorgan interactions involving the heart, kidney, and liver, as well as the comorbidities frequently associated with HF [108,109,110]. Machine learning and artificial intelligence represent the second core pillar of this framework. Advanced computational techniques, such as gradient boosting, neural networks, and ensemble stacking architectures, are particularly effective in managing the complexity and high dimensionality of multi-layered datasets. These methods enable the identification of non-linear patterns and latent relationships among clinical and molecular variables. They support the development of predictive models for outcomes, including mortality, rehospitalisation, and therapeutic response, often surpassing the performance of conventional clinical risk scores. Furthermore, the integration of data from electronic health records, implantable cardiac devices, and wearable sensors facilitates the creation of early-warning systems for impending decompensation, thereby improving proactive and personalised patient management [111,112,113].

The American College of Cardiology has highlighted that, when supported by robust data infrastructures and standardised data governance frameworks, these technologies can reduce hospitalisations and enhance patients’ quality of life [114].

**Figure 5:**
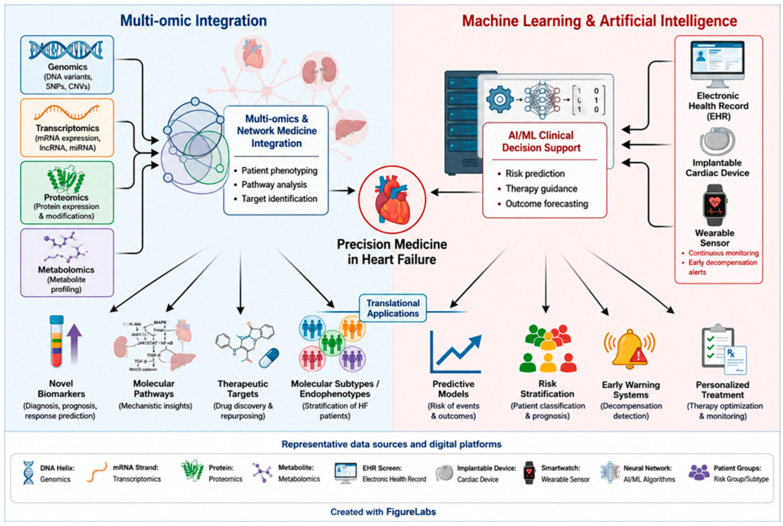
Conceptual framework for multi-omics integration in HF (AI: artificial intelligence; ML: machine learning).The blue-shaded area indicates multi-omic data integration, whereas the red-shaded area indicates machine learning and artificial intelligence applications. Icons represent the main omic layers, digital data sources, wearable/implantable devices, AI/ML algorithms, and patient risk groups/subtypes, as summarized in the legend at the bottom of the figure.

In summary, strategies for multi-layer data integration in HF are based on interconnected pillars: multi-omic integration, machine-learning-based analytics, the incorporation of electronic health records and monitoring devices, and a multidisciplinary care framework (Figure 5). When combined, these elements overcome the limitations of traditional classification schemes, facilitate the identification of clinically meaningful endophenotypes, enhance outcome prediction, and support personalised therapy, thereby establishing the foundation of precision medicine in HF.

Integrating information from multiple omics platforms not only identifies novel biological mechanisms and biomarkers but also constitutes a critical step toward more precise characterisation of the clinical forms of HF. Molecular stratification through multi-omic approaches enables researchers to move beyond classifications based solely on clinical or functional parameters, allowing for a more accurate definition of disease phenotypes. This perspective supports the identification of distinct biological profiles that correspond to different pathophysiological trajectories, ultimately informing the development of targeted diagnostic and therapeutic strategies. Notably, omics-based phenotyping has demonstrated that heart failure with reduced ejection fraction (HFrEF) and heart failure with preserved ejection fraction (HFpEF) are biologically distinct syndromes, each with unique molecular, proteomic, and genetic profiles reflecting their differing pathophysiology.

HFrEF is characterised by proteomic profiles reflecting impaired myocardial contractility and a pro-inflammatory state. This includes elevated levels of cytokines, such as IL-6 and IL-8, which correlate with a reduced ejection fraction and worsening renal function [115]. HFrEF has also been associated with multiple genetic loci, and integrating genomic, transcriptomic, and proteomic data has enabled the identification of molecular pathways linked to disease progression and early mortality [116]. Reduced activation of the ERBB2/HER2 (Human Epidermal Growth Factor Receptor 2) has emerged as a particularly relevant mechanism among these. Indeed, systems biology approaches applied to large cohorts and subsequently validated in independent studies have demonstrated that diminished ERBB2 signalling is associated with impaired cardioprotective responses. This effect appears to involve key intracellular pathways, including Phosphatidylinositol 3-Kinase/Protein Kinase B (PI3K/Akt), MAPK and Rat Sarcoma (Ras), which play central roles in regulating cell survival and the myocardial stress response. These insights have led to the investigation of targeted therapeutic strategies, such as recombinant neuregulin-1, the natural ligand of the ERBB receptors, which activates the ERBB2/ERBB4 complex. This therapy is currently in the early stages of clinical development in the United States, aiming to modulate cardioprotective signalling pathways, promote cardiomyocyte survival and proliferation, and enhance cardiac tissue repair [117]. In HFpEF, omics-based phenotyping has consistently highlighted substantial biological heterogeneity. The limited number of genetic loci identified in HFpEF indicates that it comprises a heterogeneous group of distinct pathobiological processes, rather than a single disease entity. Integrating multi-omic datasets with advanced analytical methods, including machine learning, has enabled the identification of clinically and molecularly distinct subgroups, some of which can be detected years before the onset of overt symptoms. This approach supports earlier risk stratification and the identification of high-risk clusters, often characterised by pronounced inflammatory and immune dysregulation. Deep phenotyping is essential for refining risk assessment and guiding targeted therapeutic strategies, as conventional classification based solely on left ventricular ejection fraction (LVEF) does not capture the multidimensional complexity of HFpEF. Data-driven methodologies, particularly machine learning and cluster analysis, have identified reproducible phenotypic patterns among HFpEF patients, such as metabolically obese, elderly–frail, atrial fibrillation-dominant, cardiorenal, and pulmonary hypertension/right ventricular-predominant phenotypes [118]. Each phenotype is defined by distinct pathophysiological mechanisms, risk profiles, and prognostic trajectories, underscoring the limitations of uniform treatment strategies. Collectively, these findings support a shift from an ejection fraction-centred framework to a phenotype-driven approach consistent with precision medicine principles (Figure 5). This transition would enable more personalised therapeutic strategies, improve clinical trial design and interpretation, and ultimately enhance patient outcomes. Notably, emerging evidence indicates that therapeutic responses can vary significantly across HFpEF phenotypes. For instance, patients with metabolic comorbidities, left ventricular hypertrophy, and combined systolic and diastolic dysfunction benefit more from SGLT2 inhibitors and/or Angiotensin Receptor-Neprilysin Inhibitors (ARNIs, such as sacubitril/valsartan), as reflected in reduced rehospitalisation and mortality. Conversely, subgroups such as women with atrial fibrillation, atrial remodelling, right ventricular structural abnormalities, and diastolic dysfunction may respond more favourably to calcium channel blockers [119,120].

Additionally, comorbidities such as hypertension and diabetes are associated with increased expression of tumour necrosis factor receptor superfamily (TNFRSF) biomarkers. This association highlights potentially actionable inflammatory pathways and underscores the importance of molecularly informed patient stratification in HFpEF [121,122].

Network analysis has become a valuable tool for identifying pathway convergence in omics-based phenotyping of HF. This approach enables systematic mapping of biomarker interactions and their translation into biologically meaningful pathways that differentiate HFrEF from HFpEF. Network-based analyses of HFrEF have identified key proteins, such as N-terminal B-type natriuretic peptide (NT-proBNP), growth differentiation factor-15 (GDF-15), interleukin-1 receptor type 1 (IL-1R1), and activating transcription factor 2 (ATF2), which converge on pathways related to DNA-binding transcription factor activity, cellular protein metabolism, and nitric oxide biosynthesis. In contrast, HFpEF networks are defined by hubs such as integrin subunit beta-2 and catenin beta-1, which are linked to pathways involving cytokine signalling, extracellular matrix organisation, and inflammatory responses. Integrating biomarker networks with protein–protein interaction datasets further refines this framework, revealing distinct biological signatures underlying the two phenotypes. Specifically, HFrEF is predominantly associated with processes related to cellular proliferation and metabolic regulation, whereas HFpEF is largely driven by pathways involving inflammation and extracellular matrix remodelling. This systems-level approach enables the identification of both shared and phenotype-specific pathway modules, providing deeper insight into the mechanistic heterogeneity of HFpEF and supporting the prioritisation of phenotype-tailored therapeutic targets [123].

Overall, network analysis provides a comprehensive systems biology framework for integrating multi-omic data. This approach enables more precise molecular delineation of HFrEF and HFpEF based on pathway convergence, thereby advancing the identification of actionable targets for personalised therapeutic strategies.

## 7. Translational Targets, Biomarkers, and Precision Medicine

Multi-omics studies have facilitated the identification of molecular targets that represent emerging therapeutic opportunities. One study utilised an integrated proteomics and Mendelian randomisation approach to identify novel therapeutic targets in heart failure (HF). Researchers analysed ninety circulating cardiovascular proteins across four population-based cohorts, evaluating both observational associations and causal relationships with incident HF. Eight proteins demonstrated a robust causal association with HF: higher circulating levels of Colony-Stimulating Factor-1 (CSF-1), Galectin-3 (Gal-3), and Kidney Injury Molecule-1 (KIM-1) were linked to increased risk, while elevated levels of Adrenomedullin (ADM), Chitinase-3-like protein 1 (CHI3L1), Cathepsin L1 (CTSL1), Fibroblast Growth Factor 23 (FGF-23), and Matrix metalloproteinase-12 (MMP-12) were associated with a protective effect. Of these, seven are considered druggable, indicating potential for pharmacological modulation. Notably, ADM and Gal-3 are already under investigation in clinical trials [124]. A separate large-scale multi-omics analysis, integrating genomics, transcriptomics, and proteomics, examined over 420,000 participants to identify drug targets specific to heart failure with a reduced ejection fraction (HFrEF) and preserved ejection fraction (HFpEF). This study identified 70 targets for HFrEF and 10 for HFpEF, of which 58 were not previously reported. The analysis confirmed the central role of several biological pathways in HFrEF, including the ubiquitin–proteasome system, SUMOylation, inflammation, and mitochondrial metabolism. Alterations in SUMOylation, in particular, have been linked to cardiovascular diseases such as HF. SUMOylation regulates diverse cellular processes, including transcription, DNA repair, cell-cycle progression, protein trafficking, and stress responses. It influences subcellular localisation, stability, activity, and protein–protein interactions of modified substrates. SUMOylation is a critical post-translational modification in which Small Ubiquitin-like Modifier (SUMO) proteins are covalently attached to lysine residues of target proteins via an isopeptide bond. This modification occurs through an enzymatic cascade involving E1-activating enzymes, the E2-conjugating enzyme (Ubc9 in humans), and E3 ligases, which provide substrate specificity and regulatory control. The process begins with SUMO maturation, where precursor SUMO proteins are processed by specific proteases to expose the C-terminal Gly–Gly motif. In the presence of ATP, mature SUMO is activated by the E1 enzyme, transferred to the E2 conjugating enzyme, and ultimately attached to a lysine residue on the target protein, often facilitated by an E3 ligase. SUMOylation is reversible, as SUMO moieties can be removed from substrates by the SENP (SUMO-specific protease) family of enzymes [125,126].

Among the druggable genes, IL6R, ADM, and Endothelin Receptor Type A (EDNRA) have emerged as potential therapeutic targets for HFrEF. The absence of overlap between molecular targets identified for HFrEF and HFpEF underscores the necessity of developing subtype-specific therapeutic strategies [127]. In a separate study, Yu et al. applied bioinformatics to publicly available datasets to identify diagnostic biomarkers and elucidate molecular mechanisms underlying HF. Protein–protein interaction network analysis identified Early Growth Response 1 (EGR1), EGR2, Finkel-Biskis-Jinkins (FBJ) murine osteosarcoma viral oncogene (FOS), and FBJ murine osteosarcoma viral oncogene homolog B (FOSB) as diagnostic hub genes. These genes were validated in independent cohorts using a four-gene model that demonstrated strong discriminative performance (AUC 0.775–0.877). Consequently, these genes may serve as promising biomarkers and potential therapeutic targets for HF [128].

The prognostic value of several biomarkers has been established. Specifically, Proline and Arginine-Rich End Leucine-Rich Repeat Protein (PRELP), Cytoskeleton-Associated Protein 4 (CKAP4), S100 Calcium-Binding Protein A11 (S100A11), and Annexin A1 (ANXA1) are significantly associated with all-cause mortality across HF subtypes. These markers have demonstrated high hazard ratios and have been clinically validated in the BIOSTAT-CHF cohort. They reflect underlying processes such as inflammation, extracellular matrix remodelling, and persistent cellular stress. Additionally, PRELP, Cystatin B (CSTB), and CKAP4 are associated with an increased risk of rehospitalisation, indicating a higher likelihood of adverse events and disease recurrence. Incorporating these molecular signatures into clinical practice could enhance prognostic stratification and provide more precise risk assessment than traditional biomarkers. Integrating these markers into predictive models may support personalised therapeutic decision-making, dynamic patient monitoring, and early identification of individuals requiring intensive interventions, as indicated by the recent literature [129,130].

Precision medicine strategies in heart failure (HF) depend on integrating clinical, genomic, transcriptomic, proteomic, and metabolomic data to identify molecular subtypes, prognostic biomarkers, and specific therapeutic targets. The primary objective is to advance beyond the traditional one-size-fits-all model and enable a personalised approach to diagnosis, risk stratification, and treatment. Artificial intelligence is instrumental in analysing multidimensional datasets and identifying distinct clinico-molecular phenotypes. Table 3 provides examples of targets identified through omics platforms with potential therapeutic implications in clinical practice. Patient selection is achieved through combined molecular and clinical characterisation, facilitating the identification of groups with distinct biological profiles, such as HFpEF characterised by myocardial fibrosis or inflammatory signatures, that may benefit from targeted therapies. The use of machine learning methods further supports the integration of omics and clinical data, enhancing outcome prediction and informing therapeutic decision-making (Table 3) [69,108,131].

Despite these advances, several challenges continue to impede the translation of omics discoveries into routine clinical practice. These challenges include the need for validation in large, independent cohorts, standardisation of analytical methodologies, pre-analytical variability, and the complexity of interpreting multi-omics datasets. Additional concerns involve data privacy and the current lack of robust, reproducible biomarkers suitable for widespread clinical application. Furthermore, the cost-effectiveness and accessibility of omics technologies remain significant barriers. Feasible strategies to improve clinical implementation include developing standardised, shared analytical platforms; adopting targeted, clinically actionable biomarker panels instead of full-scale omics profiling; applying artificial intelligence-assisted data interpretation tools; and establishing dedicated training programmes for clinicians and laboratory personnel. In addition, large prospective validation studies and harmonised governing frameworks are essential to improve scalability, reproducibility and economic efficiency in routine cardiovascular practice.

The American Heart Association has emphasised that full integration of omics data into cardiovascular medicine will require standardised databases, dedicated training programmes, and well-designed clinical trials to demonstrate clear clinical benefit [132,133].

## 8. Future Directions

Single-cell and spatial omics technologies are revolutionising heart failure (HF) research by enabling precise characterisation of cellular heterogeneity and intercellular interactions within cardiac tissue. Single-cell approaches facilitate the identification of discrete cellular subpopulations, such as activated fibroblasts and distinct cardiomyocyte subsets, which play critical roles in disease progression and fibrotic remodelling but often remain undetected by conventional methods. These techniques have uncovered pro-fibrotic fibroblast populations and endothelial cell subtypes linked to inflammation and adverse remodelling in failing hearts. Spatial omics integrates transcriptomic data with cell localisation, allowing gene expression patterns to be mapped directly to tissue architecture and histopathological changes. This enables the association of specific molecular perturbations with defined regions of degeneration, fibrosis, or inflammation, supporting the discovery of novel therapeutic targets and clinically relevant biomarkers. The integration of single-cell and spatial omics, increasingly supported by advanced machine learning, is rapidly advancing the understanding of pathogenic mechanisms and refining patient stratification, with significant implications for precision medicine. Specific examples of these approaches include the identification of discrete pro-fibrotic and pro-inflammatory cellular subpopulations, as well as localised transcriptional alterations associated with myocardial degeneration and fibrosis. For example, Lee et al. applied spatial transcriptomics using the GeoMx Whole Human Transcriptome Atlas to analyse myocardial samples from 44 individuals with various cardiomyopathies and controls, generating a high-resolution map of gene expression at cellular and histological levels [134]. Key findings included the identification of differentially expressed genes associated with distinct pathological processes: UCHL1 expression in cardiomyocytes was linked to degenerative changes, while endothelial expression of CCL14, ACKR1, and PLVAP was associated with fibrotic remodelling. A pro-inflammatory endothelial subtype (PLVAP^+^, ACKR1^+^, CCL14^+^) strongly associated with fibrosis was validated through multiplex immunohistochemistry and integrative analyses of single-cell and single-nucleus RNA sequencing datasets. In cardiomyocytes, downregulation of ribosomal proteins correlated with myofibrillar disarray in hypertrophic cardiomyopathy. Additionally, novel genes implicated in HF pathogenesis, including cysteine-rich secretory protein 3 (CRIP3), 6-phosphofructo-2-kinase/fructose-2,6-bisphosphatase 2 (PFKFB2), and Tax1 binding protein 3 (TAX1BP3), were identified. Collectively, these findings underscore the importance of mapping transcriptomes of specific cells and compartments to elucidate the molecular and cellular complexity of HF, potentially leading to new therapeutic targets and biomarkers for precision-based interventions.

The integration of single-cell and spatial omics technologies with advanced computational methods marks a significant advancement toward a systems-level understanding of heart failure. High-resolution transcriptomic, epigenomic, and proteomic platforms at cellular and spatial scales now generate large multidimensional datasets that reveal cellular heterogeneity, microenvironmental interactions, and dynamic molecular processes underlying cardiac remodelling. The complexity and interconnectivity of these data necessitate analytical frameworks capable of identifying biologically meaningful patterns, robust molecular signatures for prediction, and reconstructing regulatory and signalling networks involved in disease progression. Artificial intelligence and predictive modelling are central to systems cardiology, enabling the integration of multi-omic, clinical, imaging, and phenotypic data into unified computational models. These integrative approaches have the potential to transform risk stratification, enhance prognostic accuracy, and support mechanism-informed therapeutic personalisation in heart failure.

Advanced applications of artificial intelligence (AI) and predictive modelling in systems cardiology include supervised and unsupervised machine learning (ML) methods, as well as deep learning (DL) architectures such as convolutional neural networks (CNNs) and transformer-based models, for analysing clinical, omic, and imaging data. These algorithms capture complex, nonlinear relationships in high-dimensional datasets, thereby improving early detection of structural heart disease, refining risk stratification, and enhancing the prediction of cardiovascular events beyond the capabilities of traditional models based on tabular data or limited clinical variables [135,136].

Multimodal integration of clinical, genomic, transcriptomic, proteomic, imaging (electrocardiography, echocardiography, cardiac magnetic resonance, chest radiography), and environmental data enables a more granular phenotypic characterisation and advances precision cardiovascular medicine. Predictive models derived from such integrative frameworks have demonstrated superior performance compared with conventional risk scores in cardiovascular risk assessment and therapeutic personalisation [137,138].

AI-driven automated interpretation of electrocardiographic and imaging data has already demonstrated significant clinical impact, including screening for left ventricular systolic dysfunction, hypertrophic cardiomyopathy, and aortic stenosis, as well as forecasting disease progression and treatment response. Recent evidence further supports a positive effect on clinical outcomes, with studies reporting improved diagnostic accuracy, enhanced risk discrimination, and the potential reduction in adverse events through more individualised management strategies [131,139,140].

However, significant challenges persist, including the need for rigorous multicentre external validation, harmonisation and standardisation of data acquisition and analytical pipelines, improved model transparency and interpretability, and mitigation of phenotypic variability and dataset bias. These factors may otherwise limit generalisability and hinder large-scale clinical implementation.

## 9. Conclusions

Heart failure should no longer be viewed solely as a clinical syndrome classified by ejection fraction and symptom burden, but rather as a biologically heterogeneous condition sustained by interacting neurohormonal, inflammatory, metabolic, mitochondrial, and epigenetic programmes. This complexity helps explain why conventional phenotyping often fails to fully capture disease mechanisms, prognostic diversity, and differential therapeutic responsiveness.

Recent advances in genomics, epigenomics, transcriptomics, proteomics, and metabolomics have moved the field beyond descriptive pathophysiology, opening the possibility of identifying molecular endotypes, actionable biomarkers, and mechanism-based therapeutic targets. In this framework, multi-omics integration is not simply an additional layer of information, but a necessary step toward redefining HF as a stratified disease with distinct biological architectures.

The major challenge is now translational, rather than purely descriptive. Future progress will depend on rigorous external validation, standardised analytical pipelines, integration of omics with clinical and imaging phenotypes, and explainable computational models capable of supporting patient-level decision making. Precision medicine in HF will not be achieved by accumulating more molecular data alone, but by converting biological complexity into reproducible, clinically actionable stratification. This is the key step required to move from mechanistic insight to truly personalised cardiovascular care.

## Figures and Tables

**Table 1:** Key genomic and epigenetic regulators involved in heart failure pathophysiology.

Category	Regulator	Type	Mechanism of Action	Functional Impact in Heart Failure	Therapeutic Potential
**Genetic variants (common)**	GWAS loci (e.g., PRKAG2, ANKS1A, MOSPD3)	SNPs	Polygenic modulation of gene expression and pathways	Influence susceptibility, progression, and clinical heterogeneity	Polygenic risk scores (PRs) for risk stratification
**Genetic variants (rare)**	TTN, MYBPC3, FLNC, BAG3	Loss-of-function mutations	Structural and sarcomeric dysfunction	High-impact drivers of cardiomyopathy and HF	Genetic screening, family stratification
**DNA methylation**	CpG loci (56 identified)	Epigenetic modification	Promoter hyper-/hypomethylation	Suppression of oxidative metabolism; activation of glycolysis	DNMT inhibitors (e.g., RG108)
**Histone acetylation**	p300/CBP	Histone acetyltransferases	Acetylation of transcription factors (MEF2, GATA4)	Drives pathological hypertrophy	Targetable with small-molecule inhibitors
**Histone deacetylation**	HDACs	Epigenetic enzymes	Removal of acetyl groups from histones	Promotes maladaptive remodeling	HDAC inhibitors (e.g., Givinostat)
**Chromatin readers**	BRD4 (BET family)	Acetyl-lysine readers	Enhancer activation and transcriptional amplification	Sustains hypertrophic and fibrotic programs	BET inhibitors (e.g., apabetalone)
**Chromatin remodeling**	INO80 complex	Remodeling complex	Alters nucleosome positioning and TF accessibility	Rapid induction or reversal of HF phenotype	Emerging therapeutic target
**3D chromatin structure**	H3K27ac enhancer loops, HAND1	Epigenomic architecture	Rewiring of enhancer-promoter interactions	Drives transcriptional reprogramming in DCM	Targeting enhancer regulation (experimental)
**microRNAs**	miR-21, miR-133, miR-1, miR-208	Small ncRNAs	Post-transcriptional gene silencing	Regulate hypertrophy, fibrosis, contractility	Antagomirs (e.g., anti-miR-25, anti-miR-208a)
**lncRNAs**	Mhrt, Chast, Chaer, LIPCAR	Long ncRNAs	Chromatin interaction and transcriptional control	Modulate hypertrophy, autophagy, remodeling	RNA-targeted therapies, biomarkers
**circRNAs**	Multiple (miRNA sponges)	Circular ncRNAs	miRNA sequestration	Fine-tune gene regulatory networks	Stable biomarkers; delivery platforms
**Metabolic-epigenetic mediators**	Acetyl-CoA, NAD^+^, α-ketoglutarate	Metabolites	Cofactors for epigenetic enzymes	Link metabolism to chromatin state	Metabolic modulation strategies

(GWAS: genome-wide association studies, *PRKAG2*: Protein Kinase AMP-Activated Non-Catalytic Subunit Gamma 2, *ANKS1A*: Ankyrin Repeat and Sterile Alpha Motif Domain Containing 1, *MOSPD3*: Motile Sperm Domain Containing 3, SNPs: single nucleotide polymorphisms, PRS: polygenic risk scores, *TTN*: titin, *MYBPC3*: Myosin Binding Protein C, Cardiac, *FLNC*: Filamin C, *BAG3*: BCL2 Associated Athanogene 3, HF: heart failure, CpG: Cytosine–Phosphate–Guanine dinucleotide, DNMT: DNA Methyltransferase, RG108: N-Phthalyl-L-tryptophan DNA methyltransferase inhibitor, p300/CBP: E1A Binding Protein p300/CREB-Binding Protein, MEF2: Myocyte Enhancer Factor 2, GATA4: GATA Binding Protein 4, HDACs: Histone Deacetylases, BET: Bromodomain and Extra-Terminal domain protein family, BRD4: Bromodomain Containing Protein 4, INO80: INO80 Chromatin Remodelling Complex ATPase, H3K27ac: Histone H3 Lysine 27 Acetylation, HAND1: Heart and Neural Crest Derivatives Expressed 1, DCM: Dilated Cardiomyopathy, miR: microRNA, miR-21/miR-133/miR-1/miR-208: specific microRNA species, ncRNAs: non-coding RNAs, lncRNAs: long non-coding RNAs, *Mhrt*: Myosin Heavy-chain-associated RNA Transcript, *Chast*: cardiac hypertrophy associated transcript, *Chaer*: cardiac hypertrophy-associated epigenetic regulator, *LIPCAR*: Long Intergenic Non-Protein Coding RNA, Cardiac Associated, circRNAs: circular RNAs, NAD^+^: nicotinamide adenine dinucleotide oxidised form, α-ketoglutarate: Alpha-ketoglutarate 2-oxoglutarate).

**Table 2:** Major metabolic pathways and representative metabolites altered in heart failure.

Metabolic Pathway	Representative Metabolites	Direction of Change in Heart Failure	Biological Interpretation	Clinical/Biomarker Relevance
**Fatty acid metabolism (β-oxidation)**	Long-chain acylcarnitines (C16, C18), free fatty acids	↑ acylcarnitines, ↓ efficient FA oxidation	Incomplete β-oxidation due to mitochondrial dysfunction	Associated with disease severity and adverse prognosis
**Glucose metabolism/Glycolysis**	Glucose, lactate, pyruvate	↑ lactate, ↑ glycolytic intermediates	Shift toward glycolysis and reduced oxidative glucose metabolism	Reflects energetic stress and impaired perfusion
**Tricarboxylic acid (TCA) cycle**	Succinate, fumarate, malate, citrate	↑ circulating intermediates	Mitochondrial dysfunction with metabolite overflow and impaired oxidative metabolism	Succinate linked to hypoxia, inflammation, worse outcomes
**Ketone body metabolism**	β-hydroxybutyrate, acetoacetate	↑ ketone bodies	Compensatory increase in ketone utilization as alternative fuel	Correlates with disease stage and metabolic adaptation
**Amino acid metabolism**	Branched-chain amino acids (leucine, isoleucine, valine), glutamine, glycine	↑ BCAAs, altered AA profiles	Impaired BCAA catabolism and altered nitrogen balance	Predictive of insulin resistance and HF progression
**Redox and oxidative stress pathways**	NAD^+^/NADH ratio, glutathione (GSH/GSSG)	↓ NAD^+^ availability, ↑ oxidative stress markers	Redox imbalance contributing to metabolic and contractile dysfunction	Associated with mitochondrial failure and prognosis
**Lipid signaling and membrane metabolism**	Ceramides, sphingomyelins, phosphatidylcholines	↑ ceramides, altered phospholipid composition	Lipotoxicity and pro-inflammatory signaling	Strongly linked to mortality risk
**Purine metabolism**	Hypoxanthine, xanthine, uric acid	↑ purine degradation products	Increased ATP breakdown and energetic stress	Marker of severe energetic impairment

(FA: fatty acids; TCA cycle: tricarboxylic acid cycle; BCAAs: branched-chain amino acids; AA: amino acids; NAD^+^/NADH: nicotinamide adenine dinucleotide—oxidised/reduced forms; GSH: reduced glutathione; GSSG: oxidised glutathione; ATP: adenosine triphosphate; HF: heart failure). Upward arrows (↑) indicate an increase, whereas downward arrows (↓) indicate a decrease in the corresponding metabolite level or pathway activity in heart failure.

**Table 3:** Molecular pathways, omics biomarkers, and translational challenges in complex disease: from mechanisms to precision therapeutics.

Pathophysiological Pathway/Molecular Domain	Main Biological Signal	Representative Biomarkers/Omics Readouts	Therapeutic Implications	Validation/Development Status	Main Barriers to Clinical Implementation	Specific Actionable Targets (Genes/Proteins)	Clinical Indications/Patient Subgroups	Multi-omics/AI Evidence Source & Key Findings
**Cardiac Energy Metabolism**	Fatty acid oxidation ↑, Glucose utilization ↓, Mitochondrial dysfunction, NAD^+^ depletion	ACADVL, CPT1B, PPARα; NAD^+^, Acetyl-CoA, acylcarnitines; lactate; TCA intermediates (metabolomics)	Enhance metabolic flexibility; stimulate mitochondrial biogenesis; novel metabolic modulators	Advanced clinical validation (Metabolomics signatures replicated in multiple cohorts and linked to outcomes)	Limited phenotype specificity; inter-omic biological variability; assay standardization; HF population heterogeneity	PPARα, PGC-1α, AMPK, SIRT3, ACADVL, CPT1B	HFrEF, HFpEF with metabolic impairment; exercise intolerance, insulin resistance	Metabolomics + transcriptomics; AI identifies metabolic endophenotypes and predicts response to metabolic modulators
**Inflammation and Immune Activation**	IL-6, TNF-α, CRP ↑, NLRP3 inflammasome activation, immune cell infiltration	CRP, Galectin-3, ST2; IL-6, TNF-α, IL-1β; immune cell signatures (transcriptomics, proteomics)	Anti-inflammatory therapies; NLRP3 inhibitors; immune modulation; IL-1β pathway targeting	Early clinical investigation (Prospective studies and trials ongoing)	Biological heterogeneity; need for longitudinal validation; lack of patient-stratified biomarkers	NLRP3, IL1B, IL6, TNFRSF1A, TGFBR1, ST2L (IL1RL1)	HFrEF with systemic inflammation; HFpEF with immune activation; post-infection HF	Transcriptomics + proteomics; network analysis reveals immune-inflammatory endotypes and fibrosis risk
**Fibrosis and Extracellular Matrix Remodelling**	TGF-β activation, Collagen deposition ↑, Myofibroblast activation	Galectin-3, PIIINP, MMPs, TIMP-1; miR-21, miR-29; collagen turnover markers (proteomics, epigenomics)	Antifibrotic therapies; TGF-β pathway inhibitors; ECM modulation; RAS/MRA blockade optimization	Phase I/II clinical evaluation (Biomarkers tested in small clinical trials)	Lack of antifibrotic drug end-point outcome benefit; standardization of fibrosis assessment	TGFBR1, CTGF, MMP2/MMP9, COL1A1, LOX	HFrEF and HFpEF with structural remodelling; diastolic dysfunction	Proteomics + metabolomics + imaging; AI models fibrosis trajectories and predict response to therapies
**Oxidative Stress and Redox Imbalance**	ROS production ↑, Antioxidant defenses ↓, Protein oxidation, Lipid peroxidation	8-OHdG, 4-HNE, MDA; Nrf2 pathway components; oxidized proteins (proteomics, metabolomics)	Antifibrotic strategies; Nrf2 activators; redox balancing; mitochondrial protection	Early clinical investigation (Preliminary clinical evidence)	Non-specificity of biomarkers, fluctuation with acute events; analytical variability	NFE2L2 (Nrf2), SOD2, GPX4, PRDXs	HF with oxidative stress phenotype; ischemic and diabetic cardiomyopathy	Metabolomics + proteomics; AI improves prediction of oxidative stress-related outcomes
**Epigenetic and Chromatin Remodelling**	DNA methylation changes, Histone modifications, miRNA dysregulation, Chromatin remodelling	DNA methylation patterns; Histone marks (H3K27ac, H3K9me3); miR-34a, miR-133 (epigenomics)	Epigenetic drugs (HDAC, DNMT inhibitors); miRNA therapeutics; chromatin modulators	Preclinical evidence (Validation in experimental models and pilot studies)	Tissue accessibility; lack of standardization of epigenomic assays; long-term safety	DNMT1, HDAC1/2/3, EZH2, KDM6A, miR-34a, miR-133a	HFrEF and HFpEF with adverse remodelling; therapy-resistant phenotypes	Epigenomics + transcriptomics; AI identifies epigenetic endotypes and predicts therapeutic response
**Cell Death Pathways (Apoptosis, Necroptosis, Pyroptosis, Ferroptosis)**	Caspase activation; MLKL pathway; Inflammasome activation; iron-dependent lipid peroxidation	BAX, caspases, MLKL, GPX4, iron, GSH; transcriptomic signatures (proteomics, metabolomics)	Inhibitors of apoptosis/necroptosis/ferroptosis; iron chelators; anti-inflammatory strategies	Preclinical evidence (Experimental models; early human data limited)	Complexity of pathways; need for specific and safe inhibitors	BAX, CASP3/8/9, MLKL, GPX4, FTH1, ACSL4	HFrEF with cell death phenotype; post-MI remodelling; advanced HF	Proteomics + metabolomics + transcriptomics; AI links cell death signatures with outcomes
**AI and Systems Integration**	Multi-omic integration, Network modelling, Predictive algorithms, Digital phenotyping	Multi-omic signatures; Imaging + omics; Wearable/device data (omics + digital)	Precision medicine; risk prediction; personalized therapy optimization	Phase I/II clinical evaluation (Pilot studies and prototypes)	Data privacy issues; need for large, high-quality datasets; model interpretability	Multi-gene/protein signatures, network hubs; composite risk scores	All HF phenotypes; risk prediction, prognosis, therapy personalization	AI/ML + multi-omics improves prediction, patient stratification, and clinical decision support
**Therapeutic Targets (Pharmacogenomics and Precision Therapy)**	Genetic variants, Drug response pathways, Molecular target expression	Pharmacogenomic markers; Drug targets (SGLT2, ARNI, MR, neprilysin); Polygenic risk scores (genomics, proteomics)	Drug selection; response prediction; combination therapies; precision prescribing	Advanced clinical validation (Targets in clinical use; ongoing trials for new targets)	Cost; accessibility; implementation in routine practice; healthcare disparities	SLC5A2 (SGLT2), NPR1/3 (ARNI), NR3C2 (MR), ACE2, neprilysin	HF patients selected by genetic/omics profile; responders vs non-responders	Integrative genomics + proteomics + clinical data; AI predicts drug response and adverse events

(SNS: sympathetic nervous system; RAAS: renin–angiotensin–aldosterone system; AVP: arginine vasopressin; HFrEF: heart failure with reduced ejection fraction; HFpEF: heart failure with preserved ejection fraction; CRP: C-Reactive Protein; Gal-3: Galectin-3; TNFRSF: Tumour Necrosis Factor Receptor Superfamily; NLRP3: NLR Family Pyrin Domain Containing 3; NO: nitric oxide; cGMP: cyclic guanosine monophosphate; PKG: Protein Kinase G; CMD: coronary microvascular dysfunction; EndMT: endothelial-to-mesenchymal transition; NAD^+^: nicotinamide adenine dinucleotide; ROS: reactive oxygen species; BET: bromodomain and extra-terminal domain; HDAC: histone deacetylase; miRNA: MicroRNA; lncRNA: Long Non-Coding RNA; circRNA: circular RNA; AI: artificial intelligence; *NPPA*: Natriuretic Peptide A Gene; *NPPB*: Natriuretic Peptide B Gene; NPR1: Natriuretic Peptide Receptor 1; AGTR1: Angiotensin II Receptor Type 1; NR3C2: Nuclear Receptor Subfamily 3 Group C Member 2; AVPR1A: Arginine Vasopressin Receptor 1A; AVPR1B: Arginine Vasopressin Receptor 1B; PDE5A: Phosphodiesterase 5A; *IL1B*: Interleukin-1 Beta; IL-6: Interleukin-6; TNFRSF1A: Tumour Necrosis Factor Receptor Superfamily Member 1A; TNFRSF1B: Tumour Necrosis Factor Receptor Superfamily Member 1B; *LGALS3*: Galectin-3 Gene; IL1RL1: Interleukin-1 Receptor Like 1; ST2: Suppression of Tumorigenicity 2; TGFBR1: Transforming Growth Factor Beta Receptor 1; NOS3: Nitric Oxide Synthase 3; GUCY1A1: Guanylate Cyclase 1 Soluble Subunit Alpha 1; GUCY1B1: Guanylate Cyclase 1 Soluble Subunit Beta 1; EDN1: Endothelin 1; ANGPT2: Angiopoietin 2; VCAM1: Vascular Cell Adhesion Molecule 1; ICAM1: Intercellular Adhesion Molecule 1; PPARGC1A: Peroxisome Proliferator-Activated Receptor Gamma Coactivator 1 Alpha; SIRT3: Sirtuin 3; NFE2L2: Nuclear Factor Erythroid 2–Like 2; CPT1B: Carnitine Palmitoyltransferase 1B; ACADM: Acyl-CoA Dehydrogenase Medium Chain; SLC25A: Solute Carrier Family 25; TFAM: Transcription Factor A, Mitochondrial; GPX4: Glutathione Peroxidase 4; ACSL4: Acyl-CoA Synthetase Long Chain Family Member 4; FTH1: Ferritin Heavy Chain 1; RIPK1: Receptor Interacting Serine/Threonine Kinase 1; RIPK3: Receptor Interacting Serine/Threonine Kinase 3; MLKL: Mixed Lineage Kinase Domain-Like Protein; CASP3: Caspase 3; GSDMD: Gasdermin D; HDAC1/2/3: Histone Deacetylase 1/2/3; BRD4: Bromodomain Containing 4; DNMT1/3A: DNA Methyltransferase 1/3A; TET2: Ten-Eleven Translocation 2; EZH2: Enhancer of Zeste Homologue 2; KDM6A: Lysine Demethylase 6A; JMJD3: Jumonji Domain-Containing Protein 3; miR-21: MicroRNA-21; miR-29a: MicroRNA-29a; miR-133a: MicroRNA-133a; miR-208a/b: MicroRNA-208a/b; miR-499: MicroRNA-499; LIPCAR: Long Intergenic Non-Protein Coding RNA Predicting Cardiac Remodelling; *MALAT1*: Metastasis Associated Lung Adenocarcinoma Transcript 1; RNA: Ribonucleic Acid; *MYH7*: Myosin Heavy Chain 7; *TTN*: Titin; *PLN*: Phospholamban; *SCN5A*: Sodium Voltage-Gated Channel Alpha Subunit 5; *RBM20*: RNA Binding Motif Protein 20; *DES*: Desmin; *AGT*: Angiotensinogen;*KCNQ1*: Potassium Voltage-Gated Channel Subfamily Q Member 1; ATAC-seq: Assay for Transposase-Accessible Chromatin using Sequencing; RNA-seq: RNA Sequencing; ↑: increase; ↓: decrease).

## Data Availability

Data are contained within the article.
